# Chemiluminescence Biosensor for the Determination of Cardiac Troponin I (cTnI)

**DOI:** 10.3390/bios13040455

**Published:** 2023-04-03

**Authors:** Robert Tannenberg, Martin Paul, Bettina Röder, Santosh L. Gande, Sridhar Sreeramulu, Krishna Saxena, Christian Richter, Harald Schwalbe, Claudia Swart, Michael G. Weller

**Affiliations:** 1 Federal Institute for Materials Research and Testing (BAM), Richard-Willstätter-Strasse 11, 12489 Berlin, Germany; 2 Goethe-University Frankfurt, Max-von-Laue-Str. 7, 60438 Frankfurt am Main, Germany; 3 National Metrology Institute (PTB), Bundesallee 100, 38116 Braunschweig, Germany

**Keywords:** acute myocardial infarction (AMI), heart attack, emergency, diagnosis, cardiac troponin I (cTnI), biomarker, immunosensor, biosensor, chemiluminescence, luminol, peroxidase, monoclonal antibody, flow injection assay, microfluidic system, monolithic column

## Abstract

Cardiac vascular diseases, especially acute myocardial infarction (AMI), are one of the leading causes of death worldwide. Therefore cardio-specific biomarkers such as cardiac troponin I (cTnI) play an essential role in the field of diagnostics. In order to enable rapid and accurate measurement of cTnI with the potential of online measurements, a chemiluminescence-based immunosensor is presented as a proof of concept. A flow cell was designed and combined with a sensitive CMOS camera allowing sensitive optical readout. In addition, a microfluidic setup was established, which achieved selective and quasi-online cTnI determination within ten minutes. The sensor was tested with recombinant cTnI in phosphate buffer and demonstrated cTnI measurements in the concentration range of 2–25 µg/L. With the optimized system, a limit of detection (LoD) of 0.6 µg/L (23 pmol/L) was achieved. Furthermore, the selectivity of the immunosensor was investigated with other recombinant proteins, such as cTnT, and cTnC, at a level of 16 µg/L. No cross-reactivity could be observed. Measurements with diluted blood plasma and serum resulted in an LoD of 60 µg/L (2.4 nmol/L) and 70 µg/L (2.9 nmol/L), respectively.

## 1. Introduction

In our modern society with its aging population, heart diseases remain a major cause of morbidity and mortality, with 1.19 million deaths per year in 2017 and EUR 169 billion of estimated related costs for health care systems [1]. Guidelines on the prevention, diagnosis and treatment of heart diseases such as acute coronary syndrome have been published by the European Society of Cardiology (ESC) [2,3]. In the case of acute myocardial infarction (MI), cardiac troponin (cTn) is the most important biomarker for the diagnosis [3,4]. Cardiac troponin (cTn) is a protein complex consisting of troponin I (cTnI), troponin T (cTnT), and troponin C (cTnC), located in the heart muscle and is released into the bloodstream when muscle cells are damaged. The release of cTn into the blood is not exclusive to MI but can occur in various acute and chronic conditions. However, more specific to MI is a typical rise and fall of cTn levels over time. A diagnosis is based on the cTnI concentration at baseline level and changes in its concentration usually over 2–6 h, depending on the sensitivity of the assay used, as well as considering other risk factors to distinguish between chronic conditions and MI [5]. Concentrations of cTnI in the blood of healthy people vary between 1 and 50 ng/L [6,7]. They can exceed concentrations of 50–100 µg/L in the most extensive infarctions depending on the size of the injured area in patients without reperfusion [8,9]. Since troponin is a complex of three individual proteins (cTn ITC), which can be present in the blood in different fragments after an infarction, it should be possible to detect cTnI both individually and as part of smaller complexes, such as cTn IC [10,11].

The results may deviate significantly when conventional POCTs, biosensors, and laboratory-based measurements are used. Therefore, for a reliable diagnosis, the comparability of the results is of the utmost importance. Traceability to the International System of Units (SI) is the preferable way to ensure comparability of the results. For this purpose, a commutable reference or calibration material with assigned values traceable to the SI [12,13] can be used. Previous studies have provided valuable information on recombinant expression and purification of various constructs of cTn [14,15].

In order to achieve the fast and reliable detection of cTnI, a biosensor was developed within the project 18HLT10 CardioMet [16], a project funded in the framework of the European Metrology Program for Innovation and Research (EMPIR).

To date, a huge number of different biosensors for troponin have been developed. Therefore, it is not possible to discuss them in detail here. We refer instead to some excellent reviews covering this topic [17,18]. A common technique for quantifying troponin is the sandwich ELISA, which is well-established but usually a laboratory-based approach due to its heterogeneous assay principle. Chemiluminescent microparticle immunoassays (CMIA) can reach very low detection limits, down to 1 ng/L [4]. However, because of their complexity and the extensive hardware required, the devices are large and expensive [19]. Other options for quantification are sensing platforms based on surface-enhanced Raman spectroscopy (SERS). One advantage is the high sensitivity with which cTnI can be detected in the lower ng/L range [20]. Paper-based assays, such as later flow assays, are another alternative. They are cheap and easy to handle but play a minor role due to poorer detection limits and usually only semi-quantitative results. Most tests have in common that they are based on a sandwich principle, in which the analyte is bound by two antibodies addressing two different epitopes of the protein. A recently published work is based on an SPR sensor that allows a label-free detection of cTnI down to 0.5 µg/L in a buffer using a molecularly imprinted polymer [21].

Flow-based biosensors based on laser-induced fluorescence detection have previously demonstrated the detection of small molecules such as cocaine [22] and TNT [23]. However, it could be shown that blood serum and plasma are difficult matrices for detecting cTnI by fluorescence in a homogeneous assay due to heavy autofluorescence. Hence a new approach based on chemiluminescence was chosen and investigated.

**Figure 1:**
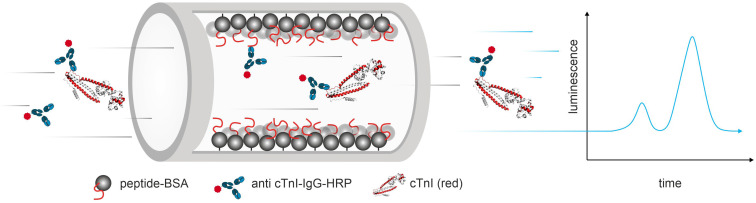
Simplified biosensor principle: The cTnI–antibody–peroxidase conjugate binds to the analyte cTnI (PDB 4Y99) and cannot interact with the peptide-functionalized column (coated with a peptide–BSA conjugate). The intensity of the chemiluminescence produced by the enzyme–substrate reaction is largely proportional to the analyte concentration in the sample.

In this work, a chemiluminescence-based immunosensor was developed for the homogeneous detection of larger molecules like cTnI, which was not demonstrated before. Due to the competitive mechanism, larger molecules pose a significant challenge in this context. Concerning fluorescence-based approaches known from the literature, the new setup led to significant miniaturization since a complex and relatively expensive optical design, including lasers, was unnecessary. The immunosensor shown in Figure 1 is based on the detection principle of chemiluminescence. This way, nonspecific background signals (e.g., stray light) and autofluorescence due to the sample matrix can be avoided.

Miniaturization is a significant advantage compared to more complex and expensive large-scale devices [24]. Furthermore, this sensor allows quasi-continuous measurements where, compared to SPR, no intermediate rinsing or regeneration steps are necessary after each sample [21]. Proven cTnI-specific antibodies [25,26] labeled with horseradish peroxidase are mixed with the sample. The mixture is then passed over an affinity column consisting of a borosilicate glass monolith functionalized with a surrogate peptide of the cTnI epitope against which the antibody is directed. Samples containing cTnI block the specific binding site of the antibody, which prevents the conjugate from binding to the peptide column and immediately passes through the detector. Subsequent light emission is caused by the enzymatic reaction of the substrate. If no analyte is present in the sample, the labeled antibody is quantitatively captured on the peptide column. Since the assay mechanism is dependent on the slow off-rate of the antibody–analyte complex, some specific consequences arise. In the optimal case, the antigen–antibody complex passes the column without dissociation. Therefore, a high excess of antibody conjugate can be used, which enhances the formation of antibody–analyte complexes, a strict prerequisite for any immunochemical technique.

In this work, a novel antibody/peptide combination has been tested to prove the applicability of this biosensor approach.

This work aimed to determine cTnI using a novel, chemiluminescence-based microfluidic biosensor. The monolithic affinity column required for the assay was chosen due to its high physical resistance and very low band broadening [27]. A new protocol for the functionalization with a peptide–BSA conjugate was established. In addition, the cTnI peptide corresponding to the antibody epitope was designed, synthesized, and subsequently conjugated to the carrier protein bovine serum albumin (BSA). This peptide–BSA conjugate avoids the need for large amounts of cTnI, which otherwise would be needed to prepare the affinity column. A large column capacity is required to reduce the requirement for regeneration steps or avoid regenerations at all.

## 2. Materials and Methods

### 2.1. Reagents, Buffers, Materials, and Equipment

The peptide Pep188-199 (CGGGDWRKNIDALSG-NH_2_, 1548.1 g/mol, 95.7% purity) was synthesized by peptides & elephants (Hennigsdorf, Germany). Bovine serum albumin (BSA) > 98% (A7906), BSA protease-free > 98% (A7030), ProClin300 (8912-U), 4-[4-(Dimethylamino)phenylazo]benzoic acid N-succinimidyl ester (SBAP, 09278-25MG-F), (3-Aminopropyl)triethoxysilane (440140), sinapic acid (85429-1G), and Mucasol (Z637203) were purchased from Sigma-Aldrich (Taufkirchen, Germany). Phosphate-buffered saline (PBS)- (1X Dulbecco’s)-Powder and tris base (77-86-1) were received from AppliChem GmbH (Darmstadt, Germany). Transparent and white flat-bottomed high-binding 96-well microtiter plates were ordered from Greiner Bio-One (Frickenhausen, Germany). Zeba™ Micro Spin desalting columns (7 kDa MWCO, 75 µL), high-sensitivity Neutravidin-HRP (31030), EZ-Link™ NHS-PEG_12_-Biotin (21312), trifluoroacetic acid 99.5% (TFA, 85183), and sodium cyanoborohydride (168552500) were obtained from Thermo Scientific (Waltham, MA, USA). PD-10 desalting columns packed with Sephadex G-25 resin (15 mL, 5 kDa MWCO) were acquired from Cytiva (Washington, DC, USA). Monoclonal anti-human troponin I antibody (Anti-h cTnI 9707 SPTN-5) was received from Medix Biochemica (Espoo, Finland). The HRP Conjugation Kit—Lightning-Link (ab102890) was from Abcam (Amsterdam, The Netherlands). The chemiluminescent substrate (SuperSignal West Pico Plus, 34580) was bought from Thermo Scientific (Waltham, MA, USA), Tween 20 (37470.01) from Serva (Heidelberg, Germany), and absolute ethanol (2246), acetonitrile 99.95% (ACN, 2697), and 150 mL filtration cups with 0.2 µm PES membrane from Th. Geyer (Renningen, Germany). PlateBlock (112500) and LowCross-Buffer (100500) were received from Candor Bioscience (Wangen, Germany). Peroxidase-conjugated AffiniPure goat anti-mouse IgG (115-035-003) and AffiniPure goat anti-mouse IgG (115-005-164) were purchased from Jackson ImmunoReasearch Europe Ltd. (Cambridge House, London, UK). Polyclonal goat anti-human cardiac troponin I (4T21/2), recombinant (rec.) human cTnI (8RTI7), and troponin I free serum (8TFS) were provided by HyTest Ltd. (Turku, Finland). Lyophilized plasma (1 g per 10 mL of ultra-pure water for reconstitution) was purchased from the German Red Cross (Berlin, Germany). 3,3′,5,5′-Tetramethylbenzidine (TMB) substrate (Seramun Blau fast 2, S100-TMB) was ordered from Seramun Diagnostica GmbH (Heidesee, Germany), and sulfuric acid (15624970) from Fisher Scientific GmbH (Schwerte, Germany). Vitrapor5 glass monoliths were purchased from Robu (Hattert, Germany) [27]. Glutaraldehyde 50% in water (111-30-8) was ordered from Merck KGaA (Darmstadt, Germany).

The microfluidic flow cell was designed and produced in-house; details are shown in the Supplementary Materials (drawing.pdf). Polyoxymethylene (POM) was purchased from Grünberg Kunststoffe GmbH (Berlin, Germany) and used for the subtractive manufacturing of the cell. Planoconvex and planoconcave lenses (49875 and 48334; 9 mm diameter; 12 mm focal length) were purchased from Edmund Optics GmbH (Mainz, Germany). Immersion oil (Immersol 518 N) was received from Zeiss (Oberkochen, Germany). For the incubation loop, a PTFE tubing with an inner diameter of 1 mm was purchased from Th. Geyer (Renningen, Germany). The camera detector QHY174M-GPS was ordered from QHYCCD (Beijing, China). The microfluidic micromixer (10000759) was acquired from Microfluidic ChipShop (Jena, Germany), the injection valve (5067–4158) from Agilent (Santa Clara, CA, USA), and a Fusion 4000X syringe pump was ordered from Chemyx (Stafford, TX, USA). Omnifix 30 mL (6303643) and 50 mL (4665914) Luer slip syringes were purchased from Braun (Melsungen, Germany), and 2 mL Luer slip syringes (7644125) from Th. Geyer (Renningen, Germany).

Matrix-assisted laser desorption/ionization time-of-flight (MALDI-TOF) mass spectrometry was performed on a Bruker Autoflex Max MS. Chemiluminescence was measured with a Synergy H1 spectrometer, and the absorbance was recorded with an Epoch2 Photometer from Biotek (Winooski, VT, USA). Data evaluation was performed with SharpCap Astro Capture Software Version 3.2.6482.0 (AstroSharp Limited, Harwell, UK) and Python 3.7 in Anaconda (Austin, TX, USA) and Origin 2018G (Northampton, MA, USA). Ultrapure water (MilliQ) was supplied by a Milli-Q Synthesis A10 system (Merck, Germany).

Most general chemicals, including ultra-pure imidazole, ß-mercaptoethanol (β-ME), NaCl, Tris, CaCl_2_, Na_2_H PO_4_, KH_2_PO_4_, IPTG, arginine, glutamic acid, antibiotics for prokaryotic cells, and chloramphenicol were purchased from Carl Roth (Karlsruhe, Germany). Ampicillin was supplied by AppliChem. Tris(2-carboxyethyl)phosphine (TCEP) was ordered from AbMole Bioscience. Tris-buffer (2 M), CaCl_2_ (1 M), and NaCl (5 M) stocks were prepared in-house using the chemicals bought from Carl Roth. Competent cells NEB5α or BL21 (DE3), T7 express or T7 express lysY/Iq were bought from New England Bio-labs. Plasmid DNA purification kits were ordered from PEQLAB Biotechnologies or Qiagen. TEV protease and PKA (protein kinase A) were expressed and purified from an in-house construct [28]. HisTrap excel, HisTrap FF, HiTrap Heparin HP columns, and HiLoad 26/600 Superdex S200 pg or S75 pg were from GE Bioscience/Cytiva. Pre-casted NuPAGE 4–12% Bis-Tris gels were purchased from Novex life technologies. PageRuler™ Unstained Protein Ladder (26614X4) was ordered from Thermo Scientific (Waltham, MA, USA). For isotopic labeling of proteins in prokaryotes, ^15^N NH_4_Cl was purchased from Sigma-Aldrich.

### 2.2. Peptide Design and Peptide–BSA Conjugation

The cTnI epitope of the monoclonal mouse antibody 9707 was obtained from the corresponding data sheet of the supplier Medix Biochemica [29] and as published previously [25]. Based on this information, the following peptide amino acid sequence (Pep188–199) with a molecular weight of 1548.1 g/mol was chosen and synthesized by peptides & elephants GmbH: CGGGD**WRKNID**ALSG-NH_2_. The highlighted sequence indicates the C-terminal cTnI epitope (amino acid residues 190–195). The peptide sequence includes an N-terminal cysteine as a conjugation option, a short glycine spacer, and an amidated C-terminus.

For the conjugation, 25 mg of BSA protease-free was dissolved in 2250 µL of 0.15 M PBS at pH 8. Then, 4.625 mg of succinimidyl 3-(bromoacetamido)propionate (SBAP) was dissolved in 250 µL of DMSO and added to the BSA with a 40-fold molar excess relative to BSA. The reaction was performed in an overhead shaker at room temperature (light protected). After 3 h, a PD MiniTrap G25 Desalting Column (with 5 kDa cutoff) was equilibrated with PBS, pH 8, and used to purify the activated protein by size exclusion chromatography. Then, 2500 µL of the eluate was pipetted into 4.5 mg of Pep188–199 (10-fold molar excess relative to eluted BSA). After overnight incubation at 4 °C, the conjugate was purified with a PD MiniTrap G25 Desalting Column (with 5 kDa cutoff), eluted with PBS pH 7.4, and stored at 4 °C. For long-term storage, 0.05% of ProClin300 was added.

The successful conjugation was confirmed by MALDI-TOF MS. Then, 10 µL of the conjugate was desalted using a MicroSpin desalting column (7 kDa MWCO, 75 µL), equilibrated with purified water, and 1 µL of the eluate was spotted on the MALDI target, and 1 µL of 3,5-dimethoxy-4-hydroxy-cinnamic acid (sinapic acid, SA, 10 mg/mL in purified water: ACN, 70:30 with 0.1% TFA) was added.

### 2.3. Column Preparation and Functionalization with Peptide–BSA Conjugate

**Figure 2:**
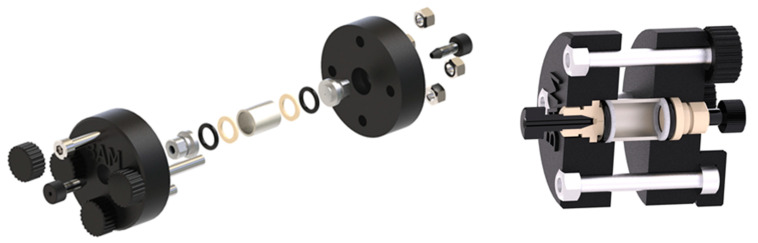
Engineering drawing of the column holder made by additive manufacturing with custom 1/16” PEEK fittings and monolithic affinity column in the center.

The column was prepared as previously described [27]. The glass monolith was glued into a titanium shell and inserted into a column holder, as shown in Figure 2.

After column insertion, some cleaning steps, silanization with (3-aminopropyl)triethoxysilane, as described in Table S1, and functionalization with the peptide–BSA conjugate were performed. First, activation was performed with 10 mL of 5% glutaraldehyde in PBS (pH 7.4) pumped through the column at 0.2 mL/min and sealed afterward. Then, the incubation was performed overnight at room temperature. After washing, the column was functionalized with 3 mL at 0.01 mL/min of peptide–BSA conjugate (around 2 mg/mL) and incubated overnight. Subsequently, 4 mL of NaCNBH_3_ (50 mM in PBS pH 7.4) was pumped through the column at 0.1 mL/min. Finally, the surface was blocked by flushing the system with 4 mL of 1% BSA (in 0.1 M Tris buffer and 50 mM NaCNBH_3_ at pH 7.4) at 0.1 mL/min, followed by purging the column with PBS buffer, including 0.1% Tween20 and 0.1% BSA, and finally with 80% of ethanol. The column was stored with 80% ethanol at 4 °C until use.

### 2.4. Troponin Expression in E. coli and cTnI Calibration

#### 2.4.1. Constructs and Molecular Cloning

Individual coding sequences of each component of human cardiac troponin protein cTnI (1M…S210; NCBI Reference Sequence: NP_000354.4), cTnT (1M…K298), and cTnC (1M…E161) were synthesized at Genscript, either with an additional NdeI and BamHI restriction sites or BsaI and XbaI restriction sites, at the N- and C-terminus, respectively. Further, all gene sequences were optimized for their expression in *E. coli*. The synthesized genes were individually subcloned into a modified pET TEV vector (a modified pET-16b vector; Novagen [28]) and a pE-SUMO vector. Subcloning troponins into the pET TEV vector was achieved by using NdeI and BamHI restriction enzymes. It provided an N-terminal His tag, followed by a TEV protease cleavage sequence (Tobacco Etch Virus, TEV) and a short linker sequence in front of the desired gene. Subcloning troponins into the pE-SUMO vector was achieved using BsaI and XbaI restriction enzymes, which resulted in an N-terminal His tag, followed by SUMO tag, in front of the desired gene. It enabled the achievement of native proteins without additional linker amino acids when cleaved with SUMO protease.

To prepare the cTn complex, cTnC and cTnT were subcloned into a dual expression vector pACYC-Duet. First, the cTnC gene constructs with restriction sites NdeI and BamHI at the N- and C-terminus were modified at the N-terminus to NcoI, retaining the BamHI at the C-terminus and cTnT gene construct with restriction sites NdeI and BamHI at the N- and C-terminus were modified at the C-terminus to KpnI, retaining the NdeI at the N-terminus. With the modified restriction sites, cTnC and cTnT were subcloned in the pACYC-Duet vector at NcoI and BamHI and NdeI and KpnI sites, respectively (Genscript).

#### 2.4.2. Protein Expression in *E. coli* and Purification

For protein expression, the recombinant plasmids were transformed into either T7 express, BL21 (DE3) or Shuffle T7 express, or T7 express lysY/Iq according to the manufacturer’s protocol (NEB). Transformed cells were plated and grown overnight on LB agar plates with either ampicillin (100 µg/mL) or ampicillin (80 µg/mL) and chloramphenicol (30 µg/mL). Single clones were picked and inoculated for performing test expressions and for preparing glycerol stocks.

#### 2.4.3. Test Expression of the cTn Constructs

The recombinant plasmids were transformed either into T7 express, BL21 (DE3), or Shuffle T7 express or T7 express lysY/Iq. With the transformants, test expressions were performed in a 10 mL LB medium with respective antibiotics. Cells were grown in a 37 °C incubator with 160 rpm, and when the cells reached an optical density of 0.6–0.8, they were induced with 1 mM IPTG (isopropyl-β-D-thiogalactopyranoside). After three hours of expression at 37 °C, cells were harvested and analyzed by SDS-PAGE. cTn recombinant constructs transformed into T7 express lysY/Iq cells have more expression of the target gene. Therefore, the cTn recombinants transformed into T7 express lysY/Iq were used for large-scale expressions.

#### 2.4.4. Large-Scale Expression

Transformed cells were inoculated into 10 mL cultures (pre-pre-inoculum) of Luria broth or ^15^N-M9 medium, with respective antibiotics, and grown for 4 h at 37 °C. From this pre-pre-inoculum, 100 mL of overnight pre-cultures in Luria broth (LB, unlabeled) or ^15^N M9 medium (for ^15^N isotopic labeling) with respective antibiotics were prepared. Cells were either directly inoculated into two liters of LB medium with antibiotics or into two liters of ^15^N M9 medium with antibiotics. Cultures were grown at 37 °C, and when the optical density reached 0.6–0.8, cells were induced with 1 mM IPTG, and expression was at 37 °C for 4–5 h. After the expression, the cells were harvested by centrifugation (4000 rpm at 4 °C for 10 min), and the cell pellets were stored at −80 °C until use.

#### 2.4.5. Protein Purification and Characterization

To purify individual cTn proteins, respective cell pellets were used, while for preparing the binary complex individual cell pellets were co-purified, and for the ternary complex cTnI with sumo tag and cTnC-cTnT cell pellets were used for the co-purification. Frozen cell pellets were thawed on ice and resuspended in their respective Ni-NTA binding buffer; i.e., lysis or loading buffer for cTnC 50 mM Tris pH 8.2, 200 mM NaCl, 5 mM CaCl_2_, 10 mM ß-ME, 5 mM imidazole was used. For cTnI and cTnT, 100 mM Tris–HCl, pH 8.2 buffer containing 1 M NaCl, 15 mM CaCl_2_, 25 mM arginine, 25 mM glutamic acid, 10 mM β-ME, and 5 mM imidazole was used. Mechanical cell disruption was performed with a microfluidizer processor, bypassing the cell suspension through the interaction chamber for 3–5 cycles at 15,000 psi. Then the lysed cells were centrifuged at 16,000 rpm for 45 min at 4 °C. The cleared supernatant separated from the insoluble pellet was loaded onto either a His-Trap HP column (Cytiva) or HisTrap Excel (Cytiva), which were equilibrated with Ni-NTA binding buffer. The loaded column was rinsed with ten-column volumes of binding buffer, followed by ten-column volumes of wash buffer (respective Ni-NTA binding buffer with 30 mM imidazole). Finally, the bound recombinant protein was subjected to a gradient elution to 100% buffer B (respective Ni-NTA binding buffer with 500 mM imidazole). Fractions containing recombinant protein were pooled after SDS-PAGE analysis and incubated with TEV protease overnight at 4 °C while dialyzing against the Ni-NTA binding buffer without imidazole. The next day inverse Ni-NTA purification was performed.

After inverse Ni-NTA purification, depending upon the purity of the protein performed, the final refinement was achieved through either size-exclusion chromatography (for cTnC, cTn IC binary, or cTn ITC tertiary complex, in the final buffer, i.e., 50 mM Tris pH 8.2, 200 mM NaCl, 5 mM CaCl2, 2 mM TCEP) or further affinity purification through heparin chromatography (cTnT, and dialyzed to the final buffer 50 mM Tris 8.2, 0.2 M NaCl, 15 mM CaCl2, 50 mM Arg, 50 mM glutamic acid, 2 mM TCEP) or affinity purification through heparin chromatography and size-exclusion chromatography (for cTnI, and the final buffer is same as for cTnT) in their respective buffers. Peak fractions of the SEC column were analyzed on SDS-PAGE for their purity before pooling the fractions. The pooled fractions of cTnC (18.6 kDa), cTnI (24.2 kDa), the binary complex cTn IC (42.8 kDa), or of the tertiary complex cTn ITC (78.3 kDa) were concentrated using either Viva spin (GE) concentrators with 10 kDa cutoff or with the regenerated cellulose (Millipore) concentrators with 10 kDa cutoff. The concentrations of the concentrated proteins were determined at the nanodrop, and smaller aliquots of the concentrated protein were stored at −80 °C. 

NMR experiments were performed at a measuring temperature of 298 K on a Bruker 950 MHz spectrometer equipped with a cryo-TXI-HCN probe. NMR samples were prepared with 5% D_2_O to lock the spectrometers, and 3-(Trimethylsilyl)-1-propane sulfonic acid sodium salt (DSS; 10 µM) was used as an internal standard for spectral referencing. NMR spectra were processed and analyzed in Topspin version 3.1 (Bruker Biospin). Sample conditions: 0.1–0.15 mM protein in 50 mM Tris pH 8.2, 200 mM NaCl, 5 mM CaCl_2_, 2 mM TCEP. ^31^P-NMR spectra were measured at 298 K on a Bruker 500 MHz spectrometer equipped with a prodigy BBO-500 S1 probe. Sample conditions: 0.1 mM protein in NEBuffer™ for protein kinases (50 mM Tris-HCl, 10 mM MgCl_2_, 0.1 mM EDTA, 2 mM DTT, 0.01% Brij 35, pH 7.5).

#### 2.4.6. MALDI-TOF-MS of Expressed Troponins

C4 ZipTips from Merck Millipore (10 µL) were used to desalt the samples according to the manufacturer’s protocol. Proteins were eluted from the tip with 2.5 µL of CHCA MALDI matrix solution (10 mg/mL, 69.9% purified water, 30% acetonitrile, 0.1% trifluoroacetic acid) directly on a spot of the MALDI target. BSA was used as a relative mass calibrator with an expected mass of 66.4 kDa.

#### 2.4.7. Calibration of cTnI by Sandwich ELISA

For the quantification of the expressed cTnI, a sandwich ELISA was used: 100 µL of the secondary capture antibody, goat anti-mouse IgG (2 µg/mL in PBS, pH 7.4), was incubated in each well of a clear, high-binding microtiter plate (MTP) overnight at 4 °C. The MTP was subsequently washed twice with 250 µL PBS and incubated for 1 h at room temperature, with 100 µL of capture antibody (2 µg/mL of monoclonal mouse anti-cTnI IgG, clone 9707, in PBS, pH 7.4, Medix Biochemica). Next, the MTP was washed three times with PBS containing 0.05 vol% of Tween 20 by an automated plate washer, and the wells were blocked with 250 µL PlateBlock (Candor Bioscience GmbH) for 1 h at room temperature. After another wash with PBST, 100 µL of the cTnI calibrator (recombinant cTnI from HyTest Ltd.) and the expressed cTnI (both diluted in LowCross-Buffer, Candor Bioscience GmbH) were pipetted and incubated for 1 h at room temperature. After a further washing step with PBST, the primary detection antibody was added. The polyclonal goat anti-cTnI IgG (HyTest Ltd.) was biotinylated by the following protocol: 2 µL of freshly prepared NHS-PEG12-Biotin (4 mg/mL in ultra-pure water) was added to 10 µL of 2 mg/mL antibody, mixed gently, and incubated overnight at 4 °C. The biotin-conjugated primary detection antibody was diluted 1:10,000 in LowCross-Buffer (Candor Bioscience GmbH), and 100 µL were transferred into the microtiter plate. After incubation for 1 h at room temperature, the plate was washed with PBST and incubated for 0.5 h with 100 µL of Neutravidin-HRP in the dark (1:30,000 dilution in LowCross-Buffer). Finally, the MTP was washed and incubated with 100 µL of TMB solution (Seramun Blau fast2). After approx. 10 min, a sufficient color change occurred, and the reaction was stopped with 100 µL of 0.25 M sulfuric acid. The absorbance was detected with an Epoch2 MTP photometer at 450 nm. A calibration curve based on commercial rec. cTnI was used to calibrate the recombinant cTnI produced in this project.

### 2.5. Antibody Labeling and Functionality Test

In order to remove preservatives and other potentially interfering compounds, 10 µL of cTnI antibody (clone 9707) with a concentration of 2.6 mg/mL (stated by the supplier) was purified with a Zeba micro spin desalting column (at a 7 kDa molecular cutoff (Thermo Scientific)), equilibrated with PBS pH 7.4, and an additional added stacker volume of 3 µL PBS pH 7.4. A commercially available HRP Conjugation Kit-Lightning-Link (Abcam) was used for the enzyme conjugation. Therefore, 13 µL of the purified antibody was added to 10 µg preactivated HRP and performed as specified by the manufacturer. An approximately two-fold molar excess of activated HRP was used relative to the antibody. The ability of the antibody-HRP conjugate to bind to the recombinant cTnI, as well as to the synthesized peptide–BSA conjugate, was tested by two approaches: a direct ELISA with peptide–BSA conjugate and a competitive ELISA with recombinant (rec.) cTnI.

For the direct ELISA and the test of the synthesized 9707-HRP conjugate, 100 µL of the peptide–BSA conjugate in PBS (pH 7.4, 0.8 µg/mL) was incubated on a high-binding MTP overnight at 4 °C and 750 rpm. After washing three times with PBST (pH 7.4 and 0.05%Tween20), further mentioned as a washing cycle, the wells were blocked with 300 µL of 1% BSA in PBST pH 7.4 and incubated for 2 h. After a washing cycle, 100 µL 9707-HRP was diluted differently in PBST-BSA (0.05% Tween 20, 0.2% BSA, pH 7.4), transferred into the wells, and shaken for 1 h at 750 rpm. After the final washing cycle, 100 µL TMB substrate was added and stopped with 0.25 M sulfuric acid.

The second approach involved a competition between the recombinant cTnI analyte and the peptide–BSA conjugate. Therefore, the peptide–BSA conjugate was immobilized on a transparent high-binding MTP (100 µL per well with 8 µg/mL). After overnight incubation at 4 °C, followed by a washing cycle, the plate was blocked with 300 µL of 2% BSA in PBS (pH 7.4) for 2 h at 750 rpm. After another washing cycle, 50 µL of different concentrations of the recombinant cTnI was added to each well, followed by 50 µL of 1:50,000 diluted 9707 monoclonal antibodies (all in PBST, 0.1% Tween20, BSA 0.1%), pH 7.4). Next, the plate was shaken for 1 h at 750 rpm, followed by a washing cycle. Next, 100 µL of peroxidase-conjugated goat anti-mouse IgG (1:50,000 diluted) was added and incubated for 1 h at room temperature and 750 rpm. Finally, the MTP was washed again, and 100 µL TMB substrate was transferred and stopped with 0.25 M sulfuric acid after approximately 10 min. The absorbance was recorded with an Epoch2 photometer at 450 and 620 nm.

### 2.6. Chemiluminescence Detection

The optical detection of the chemiluminescence was based on the commercially available camera QHY174M-GPS from QHYCCD (Beijing, China) with a CMOS sensor and a custom-designed flow cell, which was connected via T2 thread (M42x0.75). The camera used the highly sensitive Sony IMX174 front-side illuminated CMOS sensor (11.25 × 7.03 mm in size) with 2.3 megapixels (1,920 × 1,200 pixels) and a pixel pitch of 5.86 µm. In addition, the camera and the connected flow cell were shielded by a black enclosure to remove the influence of ambient light.

**Figure 3:**
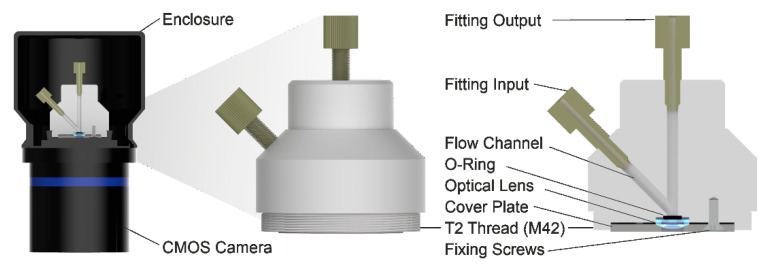
Flow cell design for chemiluminescence detection. The flow cell was manufactured from POM, and the enclosure was made from ABS.

The flow cell was designed, and precision manufactured in-house and consisted of POM; see Figure 3. The cover plate and the enclosure were additively manufactured and consisted of acrylonitrile butadiene styrene (ABS), retaining the planoconvex lens (12 mm focal length) with a diameter of 9 mm via O-ring. Both were mounted and sealed with five DIN 7991—M3x8 screws.

Microfluidic input and output tubing were guided to enter and exit the flow cell through a little gap between the CMOS camera and the enclosure (not shown). The samples, including the analyte and HRP-labeled antibodies, which were pre-mixed with the chemiluminescence substrate, were pumped through the cell. The chemiluminescence was based on a fast peroxidase-catalyzed reaction between luminol and hydrogen peroxide. This reaction emits light with a wavelength of around 425 nm. The CMOS sensor received the collected light from the flow channel focused by the planoconvex optical lens and an additional planoconcave optical lens (9 mm diameter and 12 mm focal length), which was located on top of the CMOS sensor glass cover with a few microliters of immersion oil from Zeiss (see Figure S10).

### 2.7. Fluidic Setup and Measurements

**Figure 4:**
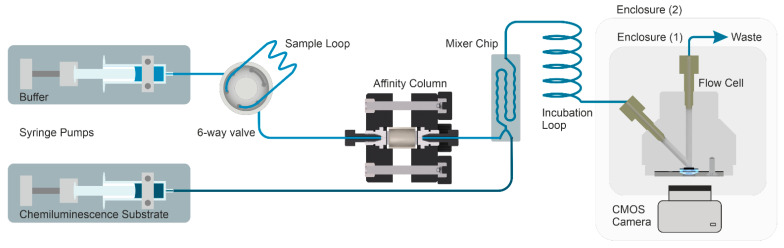
Microfluidic setup for the detection of cardiac troponin I (cTnI). The sample injection was performed by a 6-way valve and a continuous flow of running buffer and chemiluminescence substrate. After passing the peptide–BSA affinity column, the sample and substrate were combined 1:1 inside a mixer chip. This was followed by an incubation loop for preincubation and the flow cell for optical detection.

Initially, for equilibration and to remove all air bubbles inside the tubing and the flow cell, the whole microfluidic setup was flushed with filtered (0.2 µm) and degassed running buffer (PBS with 0.05% Tween 20 and 0.1% BSA). Subsequently, the running buffer and chemiluminescence substrate were injected with a high-precision dual syringe pump. The buffer flow was directed through a six-way valve, which was used to inject 500 µL samples via a sample loop to the monolithic affinity column (see Figure 4). After passing the monolithic column, the chemiluminescence substrate was blended with the sample flow with a microfluidic pearl-chain mixer, as shown in Figure S9. In this process, the sample flow was mixed 1:1 with the chemiluminescence substrate and incubated for around 11 min in an incubation loop. The chemiluminescence signal was detected via a CMOS sensor connected to the flow cell. The exit flow was collected in a waste beaker and discarded.

The CMOS camera captured a sequence of single images with an exposure time of 15 s each. The sensor was cooled to −5 °C during the whole measurement to improve the noise performance further. Due to the temperature-dependent nature of the enzymatic reaction, the temperature inside and outside the enclosure was closely monitored continuously by sensors.

### 2.8. Data Analysis

To capture the sensor data, SharpCap 3.2 software was used. Images were taken in 15 s intervals and saved as 12-bit FITC-raw files. The gain was set to zero, 2 × 2 pixel binning was selected (final image size 960 × 600 px), and the sensor temperature was set to −5 °C. Image evaluation was performed by a custom Python script (see script.py in Supplementary Materials) with a fixed region of interest (ROI) of 640 × 600 px (see Figure S11) and exported as text (txt) files. These results were evaluated using Origin 2020 (9.7.0.188). Based on the determined intensities, the areas of the sample peaks were integrated and plotted graphically.

## 3. Results

### 3.1. Troponin Peptide–BSA Conjugation and Characterization

**Figure 5:**
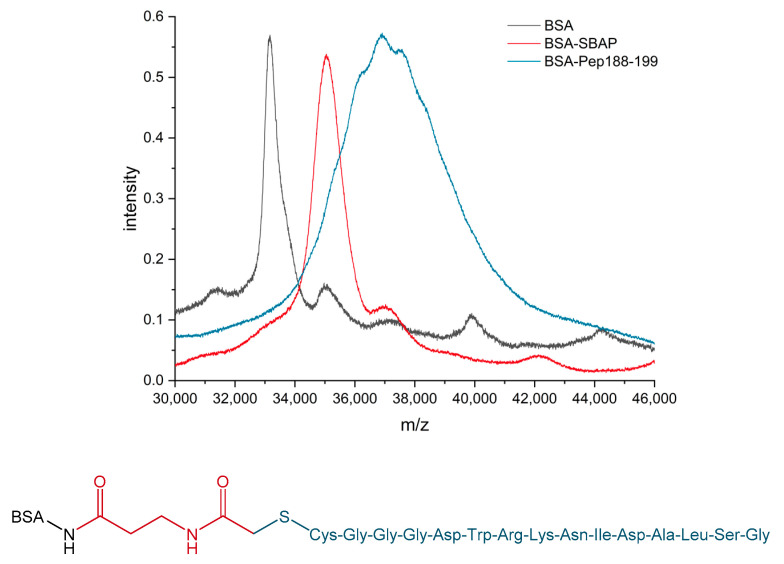
MALDI-TOF-MS of doubly charged species [M+H]^2+^ of BSA (black), BSA-SBAP (red), and final Pep188-199-SBAP-BSA conjugate (blue). For the coupling density of the final BSA–peptide conjugate, the spectrum was fitted non-linearly with a Lorentz-fit and Levenberg Marquardt iteration algorithm (fit not shown), and the peak maximum was used for the calculation. Below, a schematic structure of the conjugate is shown. The C-terminus of the peptide is amidated. Due to the shift in the spectrum towards the higher mass range, an average of about five peptides per BSA could be calculated.

A central biosensor component was the affinity column, which was functionalized with a peptide derived from a cTnI epitope. In order to be able to functionalize the column accordingly beforehand, a peptide–BSA conjugate was first prepared and characterized accordingly. For the conjugation of the peptide derived from a troponin sequence to BSA, the bifunctional crosslinker SBAP was chosen. In the first step, SBAP was conjugated to some of the primary amines of BSA (lysine side chains) via an NHS ester reaction (SBAP-BSA). In the second step, the free bromoacetyl groups on the BSA reacted with the N-terminal cysteines of the Peptide188-199. The conjugate (Pep188-199-SBAP-BSA) was analyzed by MALDI-TOF-MS. Approximately twenty molecules of SBAP and five peptides per BSA were bound on average (Figure 5).

### 3.2. Expression of cTnI in E. coli and Adjustment of the Resulting Stock Solution by ELISA

#### 3.2.1. Expression of Human Cardiac Troponins in *E. coli*

In order to obtain cardiac troponins in sufficient quantities, they were expressed in a well-established bacterial expression system. Individual subunits of the cTn were subcloned into the in-house modified pET32 inducible expression vector (see Figure S1), which facilitated the addition of a TEV protease cleavable N-terminal His tag to the target protein in *E. coli* (see Figure S2). At first, each troponin subunit was transformed into T7-express competent cells. Then, test expressions were performed with the transformants on a small scale for which we could observe overexpression of the individual troponins, respectively. However, the expression levels were not consistent upon repetition. Therefore, each troponin subunit was expressed in different competent cells, such as BL21 (DE3) or T7 express or T7 express lysY/Iq, to achieve tight regulation on the basal expression of the target gene. The test expressions of these various transformants performed in small volumes showed that the individual expression of all three troponins was highest in the T7 express lysY/Iq cells with reproducible results. Henceforth, we used T7 express lysY/Iq cells for all scaled-up expressions.

#### 3.2.2. Purification and Characterization of Recombinant Cardiac Troponins

The purification of cardiac troponins was performed from the cell pellet obtained by the overnight expression performed at 20 °C after cold shock and induction with 1 mM IPTG. Surprisingly, the yield of the protein after affinity chromatography was significantly lower for cTnI and cTnT, contrary to the test expression yields of the respective proteins. Therefore, the expression of cTnI and cTnT proteins was carried out at 37 °C for 4–5 h, which notably improved expression yields. For cTnI purification after the first Ni-NTA affinity chromatography, we observed precipitation of the protein upon removal of the fusion tag (and dialysis to remove imidazole) due to its hydrophobic nature. Therefore, we supplemented the buffers with arginine and glutamic acid, which prevented protein aggregation and precipitation to a larger extent [30]. We used similar buffer conditions for cTnT purification that were unnecessary for cTnC protein purification. In the protein purification process, we could achieve active cTnI protein in native conditions to ~1–2 mg, active cTnC protein in native conditions to ~20 mg, and cTnT to ~10 mg per liter culture. The final products were further characterized by SDS-PAGE, and bands appeared according to the molecular size before and after the cleavage with TEV protease, shown in Figure S2.

For biosensor development, recombinantly produced cTnI was used. In order to assess that the recombinant cTnI was folded correctly, we performed size-exclusion chromatography (SEC) and 2D NMR analysis using ^15^N-isotope enriched protein. Formation of the binary (cTn IC) and ternary (cTn ITC), as confirmed by the SEC profile (shown in Figure S3A), suggested that the protein was active and well folded. Further, the successful formation of the cTn IC complex was investigated by recording 2D NMR, ^1^H, ^15^N-HSQC spectrum of the proteins. An overlay of the ^1^H, ^15^N-HSQC spectra of free cTnI and the cTn IC complex revealed significant changes in the amide chemical shifts, indicating complex formation (Figure S3B,C).

#### 3.2.3. Calibration of the Stock Solution of Recombinant cTnI by ELISA

**Figure 6:**
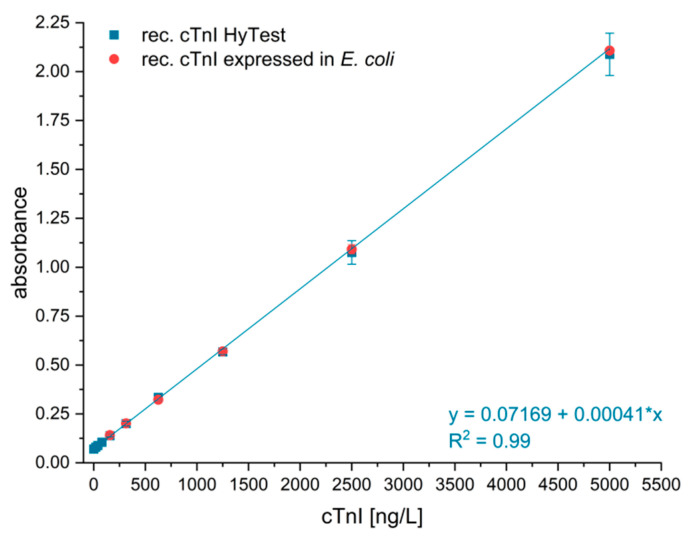
Quantification of cTnI expressed in *E. coli* using commercially available cTnI (Hytest 8RTI7) was used to calibrate the sandwich ELISA, as recommended by the supplier [31].

Recombinantly produced cTnI expressed and purified from *E. coli* was used for all biosensor measurements. A calibration with commercial recombinant cTnI was performed, and a sandwich-ELISA was established. This way, the concentration of the stock solution was determined and used for the biosensor measurements (Figure 6).

### 3.3. Performance of Peptide–BSA Column and Blank Measurement

To confirm the functionality of the peptide–BSA conjugate and the activity of the antibody–HRP conjugate, cTnI samples were analyzed with a direct and competitive indirect ELISA, shown in Figure S5A,B. Optimal CMOS sensor parameters, such as active cooling and incubation times for the chemiluminescence signal development, have previously been tested and are shown in Figures S6 and S7.

**Figure 7:**
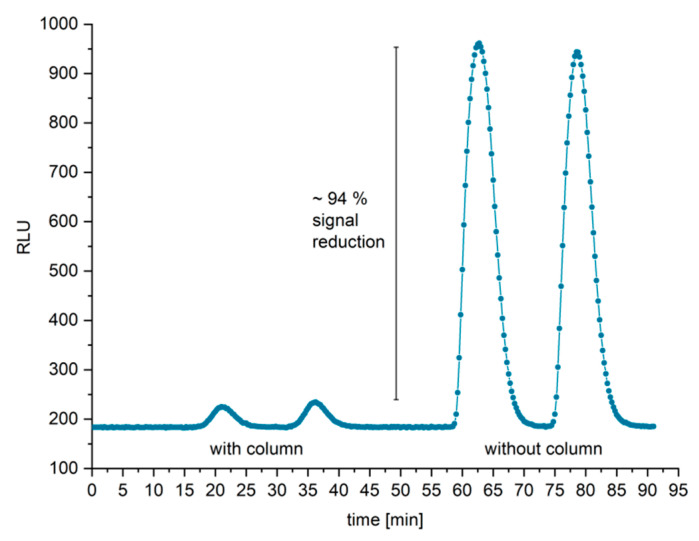
Performance test of the functionalized Pep188-199-SBAP-BSA column and the antibody–HRP conjugate (clone 9707). Injection of blank samples with and without affinity column resulted in a signal reduction of around 94% by applying a flow rate of 0.2 mL/min for each syringe pump. The signal reduction is an indication of conjugate purity and antibody activity.

The performance of the functionalized peptide–BSA affinity column and the 9707–HRP conjugate were tested by the injection of the highly diluted reagent (1:60,000). In addition, the relative chemiluminescence intensity was measured with and without an inserted affinity column, as shown in Figure 7.

**Figure 8:**
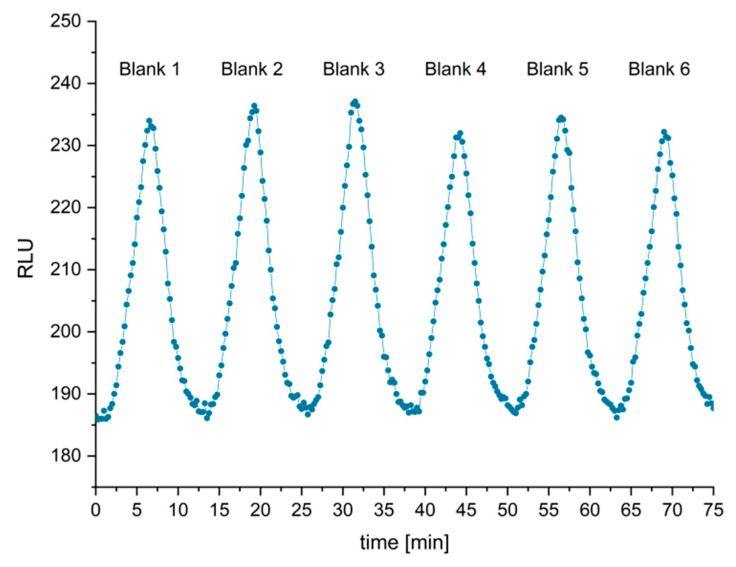
The injection of a sample series with six blanks achieved a standard deviation of 4.4% of the peak area.

The signal difference provides information about the completeness of the antibody binding with the affinity column. By inserting the affinity column, the signal was reduced by 94%, which means that this fraction of the antibody–HRP conjugate was bound to the peptide–BSA column. Six percent of the conjugate was not trapped, which might have been caused by inactive bioconjugates, non-conjugated HRP, or kinetic limitations. This fraction might be further reduced, e.g., by additional purification steps or lower flow rates. Subsequently, the antibody–HRP conjugate was injected six times into the column, shown in Figure 8. A stable baseline was obtained, and the peak area of each blank was integrated, resulting in a relative standard deviation of 4.4%.

### 3.4. Exploration of the Measurement Range

**Figure 9:**
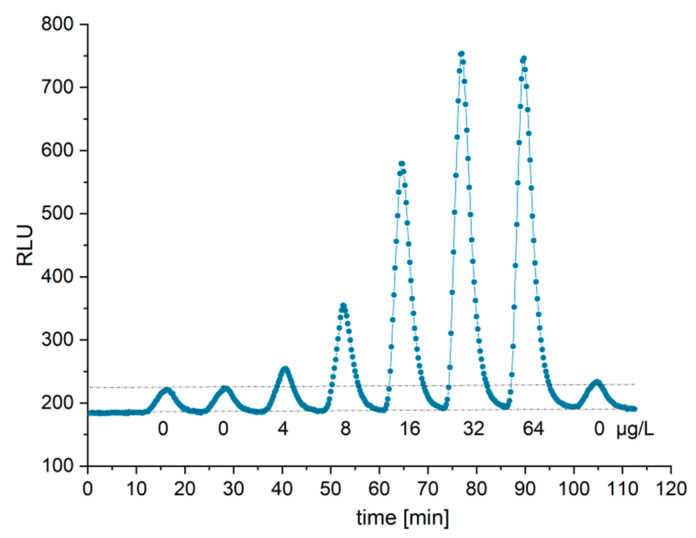
Measurement series of cTnI in a concentration range between 0 and 64 µg/L and estimation of the dynamic range (see Figure S13).

First, the measurement range of the biosensor setup was examined by injecting different concentrations of recombinant cTnI diluted in PBST-BSA buffer (0 to 64 µg/L), shown in Figure 9.

Over the whole measurement time, constant baseline and blank values were obtained. The chemiluminescence signal increased asymptotically with increasing analyte concentration up to 30 µg/mL and reached saturation. The cTnI sample with the lowest concentration, which resulted in a positive signal, was 4 µg/L.

### 3.5. Test of Specificity (Cross-Reactivity)

**Figure 10:**
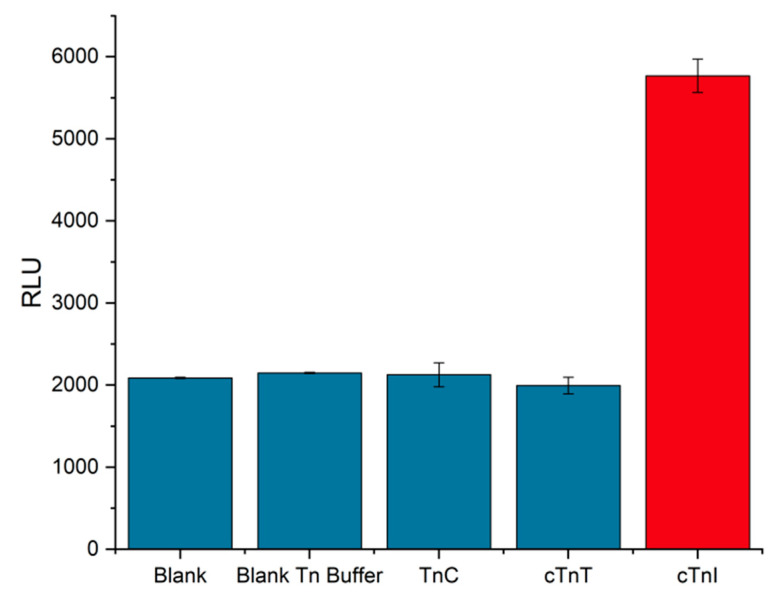
Antibody specificity test against cTnI: The biosensor detected cTnI (red column). No cross-reactivity with cTnT, cTnC, or any influence of the sample buffer was observed at 16 µg/L. Chemiluminescence was quantified by integration of the peak area. Error bars represent one standard deviation.

To ensure the antibody specificity and to rule out interference of the analyte storage buffer, different samples were tested in duplicates: cTnC, cTnT, cTnI, and the sample buffer. In addition, the blank (running buffer) was also measured as a reference (see Figure 10).

For the specificity test of the biosensor, each cTn species was diluted to 16 µg/L. No interference with the sample buffer was observed. For all samples but the cTnI, chemiluminescence signals at the background level were obtained, the same as for the negative controls.

### 3.6. Determination of LoD and LoQ for cTnI Detection in PBST-BSA Running Buffer

**Figure 11:**
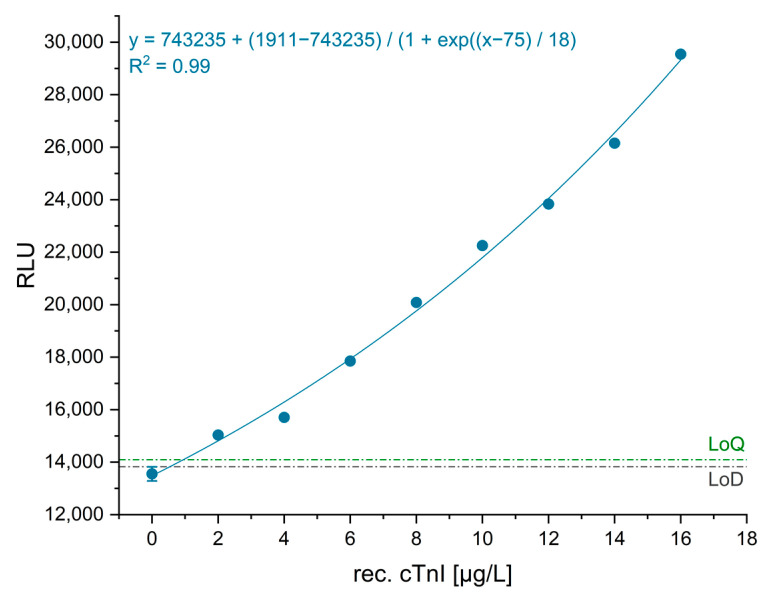
Calibration curve for troponin I in the range of 0 to 16 µg/L and calibration curve after peak integration. The error bar of the blank is shown as 3 standard deviations. The sensor data are shown in Figure S12.

The LoD and the LoQ of the biosensor were investigated in more detail. Therefore, various concentrations of cTnI were measured in a series, ranging from 0 to 16 µg/L, as shown in Figure 11. From this calibration curve, an LoD of 0.6 µg/L (3s) and an LoQ of 1.0 µg/L (6s) could be calculated.

### 3.7. cTnI Measurement in Human Plasma and Serum

**Figure 12:**
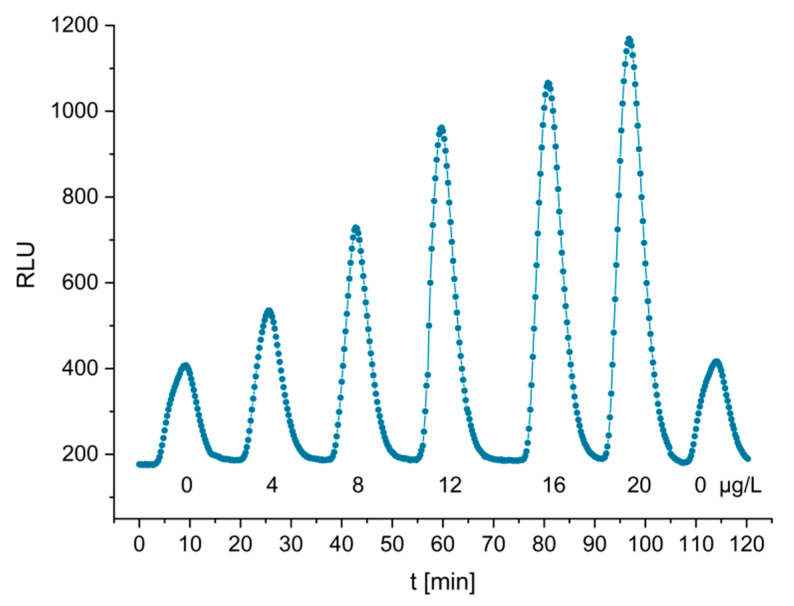
Measurement of human plasma, diluted 1:40 in running buffer and spiked with rec. cTnI in a range between 0 and 20 µg/L. The dilution factor is not included in the given concentrations.

Finally, the biosensor was tested with relevant blood matrices. Recombinant cTnI was spiked into human plasma with a defined troponin concentration and human serum. Blood plasma was not depleted of cTnI and was additionally spiked with it. However, this was considered in the blank measurements of the plasma, shown in Figure 12. On the other hand, the blood serum from HyTest was cTnI-depleted. The samples were diluted 40-fold with the running buffer to reduce matrix effects.

**Figure 13:**
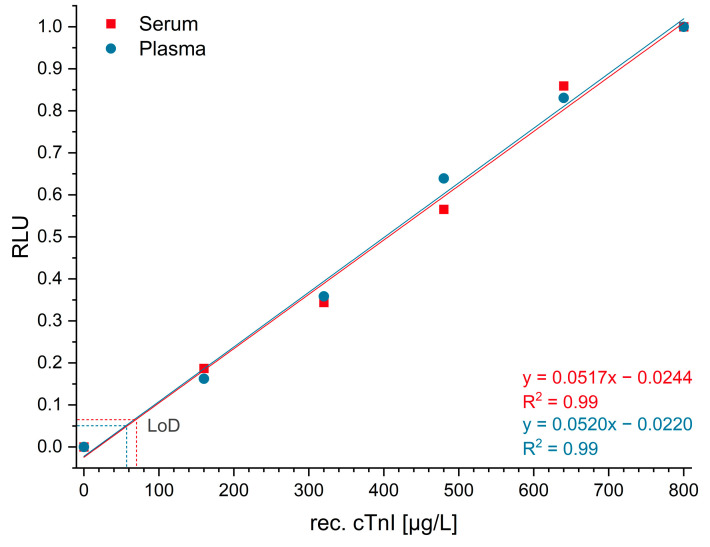
Linearity of the cTnI detection in spiked human serum and plasma. Limits of detection (LoD) of 70 and 60 µg/L for serum and plasma, respectively, have been calculated.

The calibration lines for cTnI in serum and plasma are shown in Figure 13. With a dilution of 1:40, no concentration-dependent matrix effects were observed. The experiment covered a range between 0 and 800 µg/L of spiked recombinant cTnI. LoDs of 70 µg/L for serum and 60 µg/L for plasma were calculated based on the standard deviations of the blank values of each dilution series, including the 40-fold dilution factor.

## 4. Discussion and Conclusions

This work presents a proof-of-concept of a chemiluminescence-based biosensor for the monitoring of cTnI.

For the biosensor measurements, the samples were mixed with an antibody–peroxidase conjugate, measured quasi-continuously via a microfluidic setup, and fitted to a cooled CMOS-based detection system. Most of the introduced antibody–peroxidase conjugate was trapped on the antigen-coated affinity column and hence was not detected in the subsequent chemiluminescence step. Binding sites of the antibody–peroxidase conjugate, which were bound to the analyte cTnI, were not trapped and therefore exited the affinity column without any retention. The column eluate was mixed with a high-sensitivity chemiluminescence substrate and entered the detection flow cell. The emitted light could be continuously detected with a cooled CMOS camera. This setup allowed the selective measurement of cTnI within 10 min. Fluorescence-based detection is barely suitable for high-sensitivity detection since blood serum or plasma usually shows heavy autofluorescence even in the far-red spectral range and hence leads to a very high background (data shown in Figure S13). Therefore, a novel flow cell prototype was designed, allowing continuous and sensitive chemiluminescence detection. As a label, the enzyme horseradish peroxidase (HRP) was covalently linked to a target-specific antibody and used with a luminol-based chemiluminescence substrate. HRP catalyzes the reaction of H_2_O_2_ with luminol, resulting in efficient light emission (around 425 nm). Measurements for the characterization of the system showed low detection limits of 0.6 µg/L (23 pmol/L) in buffer.

Real sample matrices, such as blood serum and plasma, are challenging due to their highly complex composition. In addition, serum and plasma samples seem to suppress even luminol chemiluminescence to some degree [32,33,34]. Hence, the dilution of those matrices is recommended. We decided to use a 40-fold dilution in buffer, which led to limits of detection of 60 µg/L for plasma and 70 µg/L for serum. Compared with high-sensitivity immunoassays [4,35] with LoDs in the range of only a few ng/L, some improvements are necessary. Based on the literature, it is known that this assay format would benefit from monovalent binders, such as Fab fragments or nanobodies [36,37,38,39]. A binder of the highest possible affinity would be desirable in all cases. Those might be obtained by affinity maturation of existing recombinant antibodies [40] or advanced library selection methods [41]. More enzymes also should be explored, which might show better compatibility with human plasma or serum. In any case, a lower sample dilution would proportionally contribute to better sensitivity.

A striking advantage of the sensor presented here is its simple and inexpensive design compared to other more complex and complicated optical methods, such as SPR [21], fluorescence, or electrochemiluminescence [17,18]. Other advantages of the setup are the small size and the inherent multiplexing ability to expand the approach to a set of biomarkers of interest. Furthermore, due to the partially homogeneous assay design, washing steps are unnecessary and therefore have short response times. Finally, it should be mentioned that the high capacity of the affinity column avoids any regeneration steps, which are usually necessary for other biosensor designs. In the next development step, continuous plasma sampling could be envisaged. This would be a big leap in abandoning the one-time measurements, whether a laboratory-based system or a quick test.

## Data Availability

Technical drawing of the flow cell: drawing.pdf; Python script for data evaluation: script.py, including corresponding txt-files: hotpixels_960_600.txt and roi_region_x_y.txt and eleven test FITC-files can be found in the Supplementary Materials.

## Supplementary Materials

The following supporting information can be downloaded from: https://www.mdpi.com/article/10.3390/bios13040455/s1, Figure S1: Human cardiac troponin constructs and expression. Figure S2: SDS-PAGE of all three troponin subunits (cTnI, cTnT, and cTnC). Figure S3: Human cardiac troponin binary and ternary complexes. Figure S4: MALDI-TOF-MS of rec. cTnI, cTnT, and cTnC expressed in *E. coli* and their expected mass: 24.2 kDa for cTnI, 35.9 kDa for cTnT, and 18.6 kDa for TnC. Figure S5: 9707-HRP antibody binding against Pep188-199-SBAP-BSA conjugate (**A**) and 9707 antibody competition between the Pep188-199-SBAP-BSA conjugated and the expressed rec. cTnI (**B**). Figure S6: Investigation of the noise behavior of the CMOS under various conditions. Figure S7: Relative luminescence unit (RLU) measurement of three different sample matrices (PBST-BSA, human serum 1:40, and human plasma 1:40). Figure S8: Drawings of the flow cell made by subtractive manufacturing and the enclosure. Figure S9: Drawing of the pearl-chain mixer chip (658) ordered from microfluidic ChipShop. Figure S10: Top view QHY 174 mono CMOS with a planoconcave lens positioned on top of the protective glass of the separate CMOS chamber. Figure S11: Images of the CMOS with 2 × 2 pixel binning resulting in an image size of 900 × 600 px. Figure S12: Calibration of recombinant troponin I in PBST-BSA in the lower concentration range. Figure S13: Exploration of the measurement range of rec. cTnI. Figure S14: Autofluorescence measurement of blood plasma with the previously described biosensor setup. Table S1: Preparation of the Pep188-199-SBAP-BSA column.
