# Supplementary material for: Chemiluminescence Biosensor for the Determination of Cardiac Troponin I (cTnI)
Source: Biosensors (Basel). 2023 Apr 3;13(4):455. doi: 10.3390/bios13040455 (PMC10136549; doi:10.3390/bios13040455)
Supplement: Supplementary file 1 [file biosensors-13-00455-s001.zip › biosensors-2237272-supplementary.docx]

Supplementary Materials

Chemiluminescence Biosensor for the Determination of
Cardiac Troponin I (cTnI)

Robert Tannenberg ^1^, Martin Paul ^1^, Bettina Röder ^1^, Santosh L. Gande ^2^, Sridhar Sreeramulu ^2^, Krishna Saxena ^2^, Christian Richter ^2^, Harald Schwalbe ^2^, Claudia Swart ^3^ and Michael G. Weller ^1^*

^1^ Federal Institute for Materials Research and Testing (BAM), Richard-Willstätter-Strasse 11, 12489 Berlin, Germany; robert.tannenberg@bam.de (R.T.); martin.paul@bam.de (M.P.); bettina.roeder@bam.de (B.R.)

* Correspondence: michael.weller@bam.de (M.G.W.); Tel.: +49-30-8104-1150

^2^ Goethe-University Frankfurt, Max-von-Laue-Str. 7, 60438 Frankfurt/M, Germany; gande@nmr.uni-frankfurt.de (S.G.); sridhar@nmr.uni-frankfurt.de (S.S.); saxena@em.uni-frankfurt.de (K.S.); ric@nmr.uni-frankfurt.de (C.R.); schwalbe@nmr.uni-frankfurt.de (H.S.)

^3^ National Metrology Institute (PTB), Bundesallee 100; 38116 Braunschweig, Germany; claudia.swart@ptb.de (C.S.)


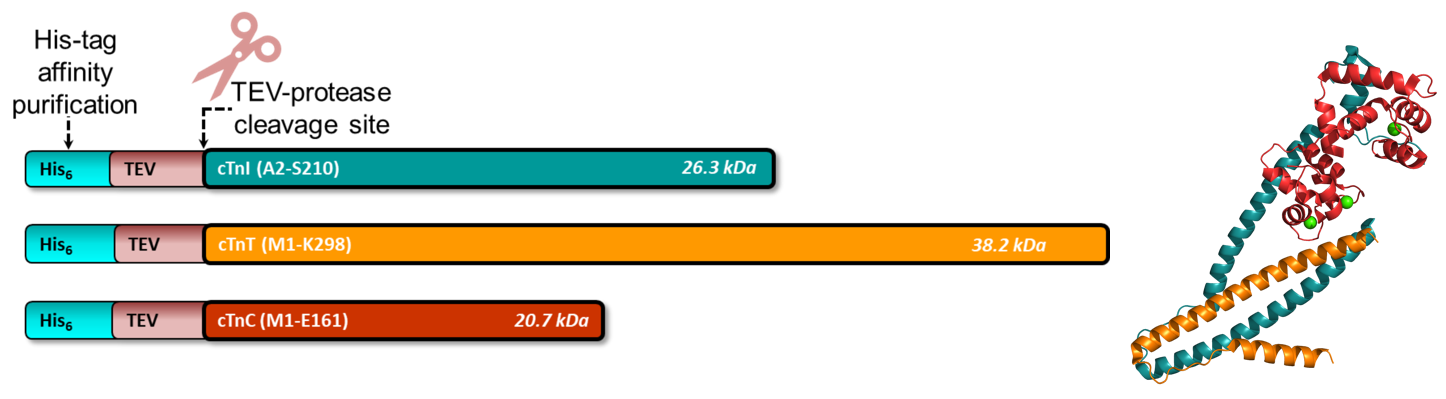
Characterization and functionality test of cTn expressed in *E. coli*


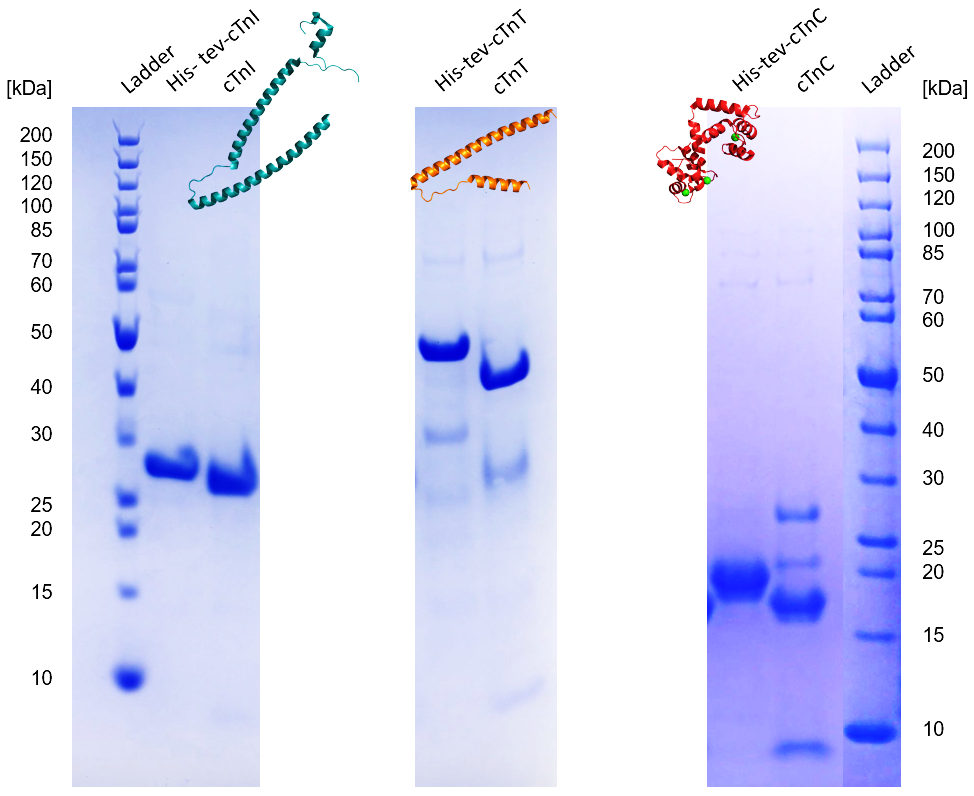
**Figure S1.** Human cardiac troponin constructs and expression. PDB deposited crystal structure of the core domain of the human cardiac protein (4Y99), shown as a reference to ease perception and correspond to the color code of the different constructs designed, intended to be expressed in the prokaryotic system (*E. coli*).

**Figure S2.** SDS-PAGE of all three troponin subunits (cTnI, cTnT, and cTnC) were recombinantly expressed in *E. coli* and purified with His fusion tag, which could be removed with the TEV protease.


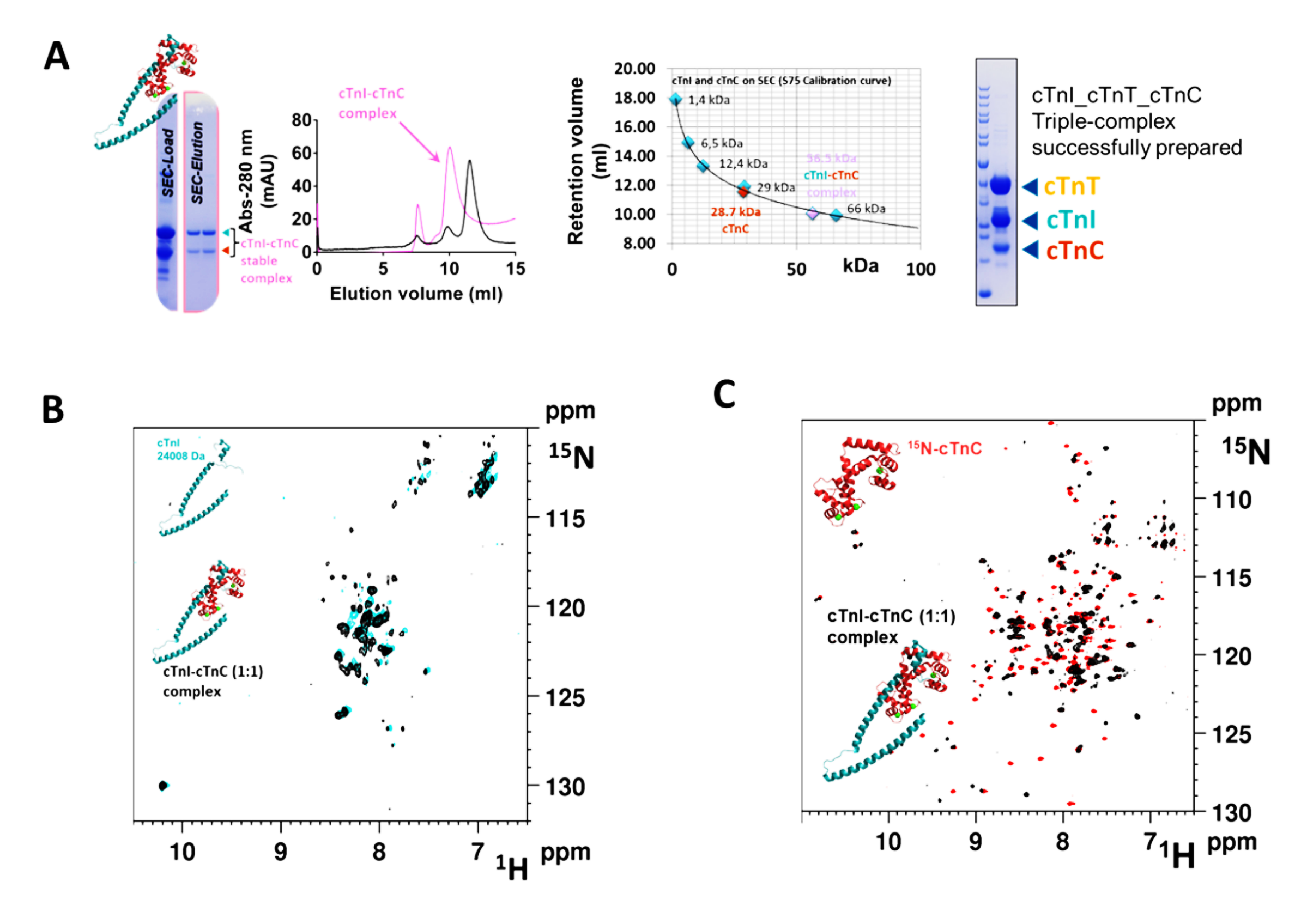


**Figure S3.** **A)** Human cardiac troponin binary and ternary complexes. cTnI and cTnC could form a stable binary complex purified on size-exclusion chromatography (SEC). Fractions of the SEC chromatography with the binary complex were shown on the SDS-PAGE (left). The chromatograms of the binary complex in the middle show a 1:1 ratio of cTnI and cTnC (in black) and with an excess of cTnI (in pink), and the graphical picture shows the arbitrary molecular weight of the binary complex with reference to the standard molecular weight markers of the SEC 75 tricon (middle). Purified cTn ternary complex on the SDS-PAGE (right). **B**) ^1^H,^15^N TROSY HSQC spectra (950 MHz) of the ^15^N-cTnI (apo, cyan spectrum) and cTnC-cTnI binary complex (holo, black spectrum). Inset: crystal structure of cTnC or cTnI-cTnC complex. The spectra were acquired at 298 K in 50 mM Tris pH 8.2, 200 mM NaCl, 5 mM CaCl_2_, and 2 mM TCEP. **C)** ^1^H,^15^N TROSY HSQC spectra (950 MHz) of the ^15^N-cTnC (apo, red spectrum) and cTnC-cTnI binary complex (holo, black spectrum). Inset: crystal structure of cTnC or cTnI-cTnC complex. The spectra were acquired at 298 K in 50 mM Tris pH 8.2, 200 mM NaCl, 5 mM CaCl_2_, and 2 mM TCEP.


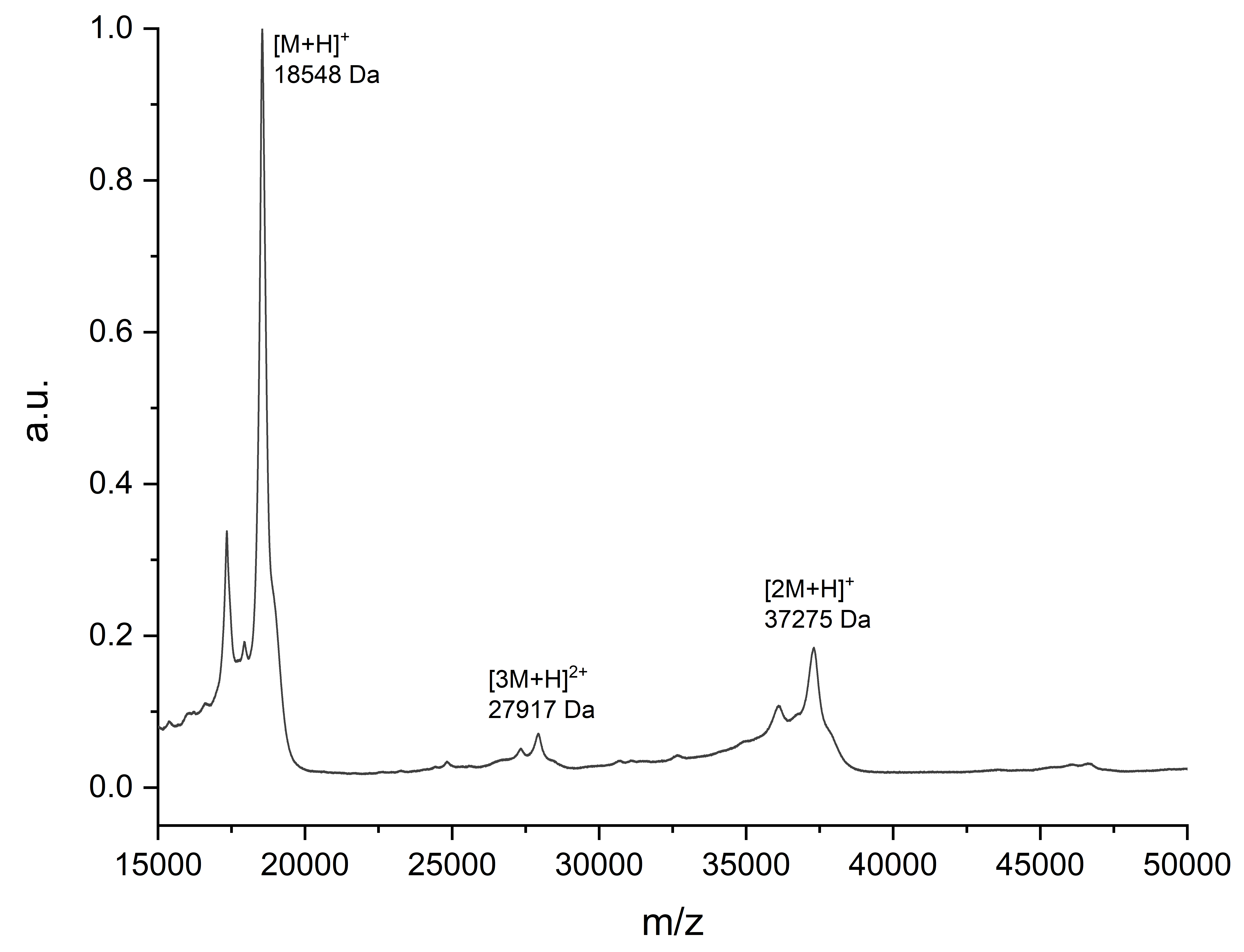

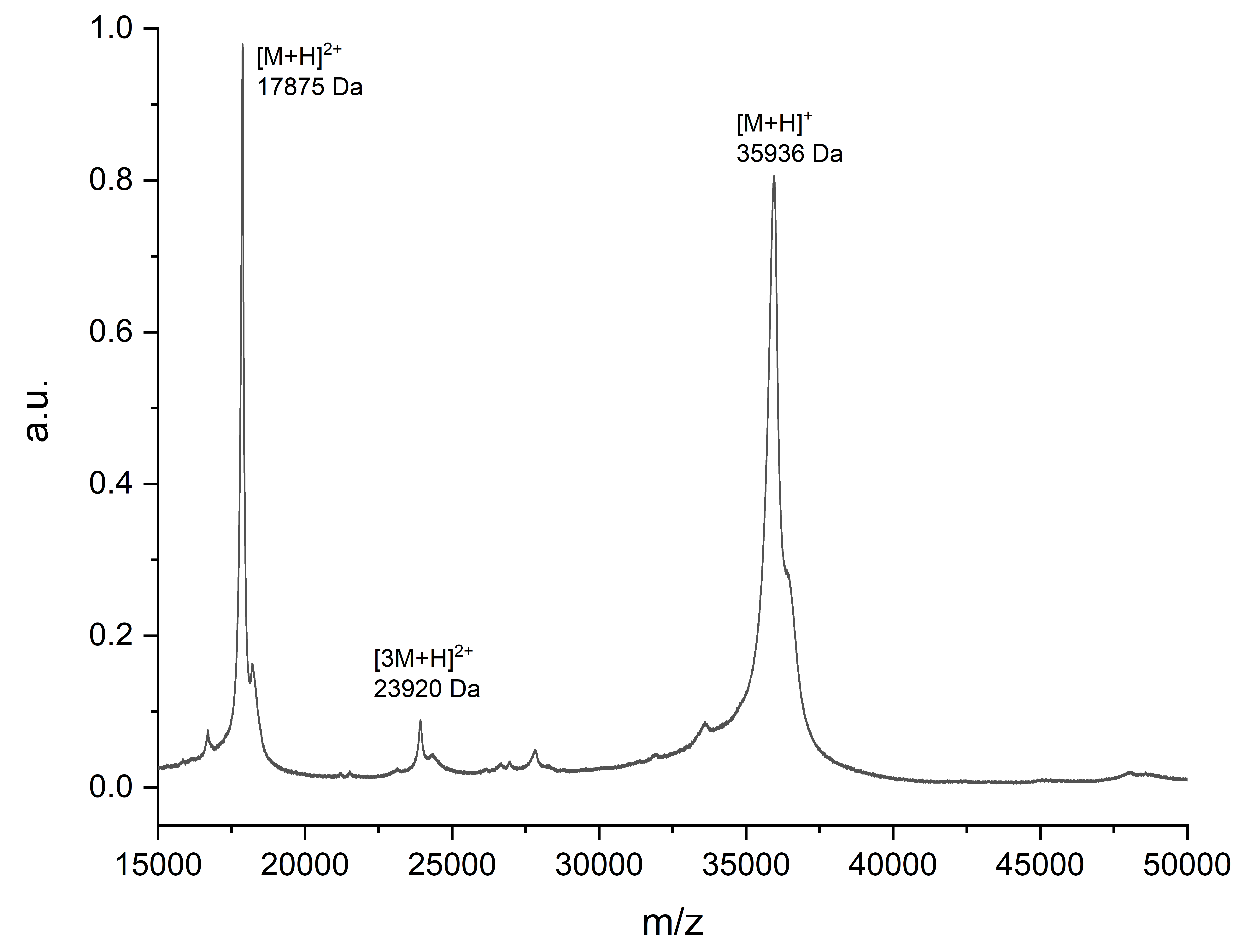

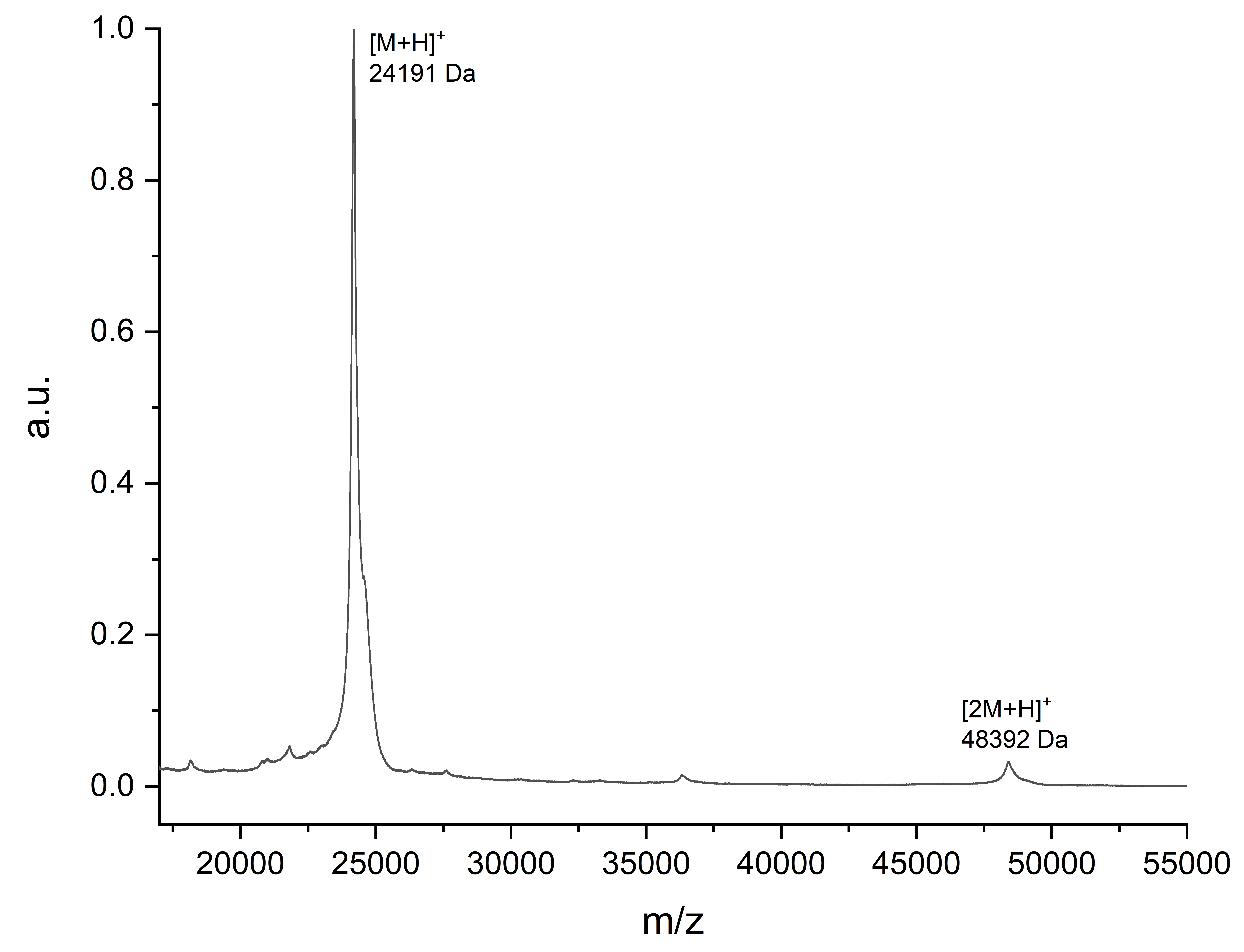
MALDI-TOF mass spectra of recombinant cTnI, cTnT, and cTnC

Figure S4. MALDI-TOF mass spectra of recombinant cTnI, cTnT, and cTnC expressed in *E. coli* and their expected mass: 24.4 kDa for cTnI, 35.9 kDa for cTnT, and 18.6 kDa for TnC.

Preparation of the affinity column

For the column functionalization, the raw columns were treated as described in Table S1. After the affinity coating, the column was stored under 80 % ethanol at 4°C and remained stable for several months.

**Table S1**. Preparation of the BSA-Peptide-188-199 column.

| **Reagent** | **Volume [ml]** | **Flow rate [mL/min]** |
| --- | --- | --- |
| Ultra-pure water | 10 | 1 |
| 10% Mucasol | 10 | 1 |
| Ultra-pure water | 10 | 1 |
| KOH 10 wt. % | 10 | 1 |
| Ultra-pure water | 20 | 1 |
| EtOH | 10 | 0.5 |
| 10 % APTES in EtOH (99%) | 10 | 0.1 |
| EtOH (99%) | 20 | 0.5 |
| Ultra-pure water | 20 | 1 |
| 5 % Glutaraldehyde in PBS pH 7.4; storage overnight at room temperature | 10 | 0.2 |
| EtOH | 20 | 0.5 |
| PBS pH 7.4 | 30 | 0.5 |
| Pep188-199-SBAP-BSA Conjugate in PBS pH 7.4 (2 mg/mL); storage for 40 h at RT | 3 | 0.01 |
| 50 mM NaCNBH3 in PBS pH 7.4 | 10 | 0.1 |
| PBS pH 7.4 | 10 | 1 |
| PBST(0.1% Tween20)-BSA(0.1%); pH 7.4 | 40 | 1 |
| Ultra-pure water | 20 | 0.5 |
| 80 % EtOH (Storage at 4 °C) | 10 | 0.5 |

Antibody labeling and functionality test

In order to verify the functionality of the antibody-HRP conjugate (clone 9707) and the ability to bind the peptide-BSA conjugate, two different approaches have been used. First, the conjugate was immobilized on a microtiter plate and incubated with varying concentrations of the 9707-HRP antibody. As a negative control, plain BSA was immobilized separately. After the incubation with TMB substrate, the absorbance was measured, as shown in figure S5, A. It could be demonstrated that the HRP-labeled antibody binds the peptide, and signals can be measured down to the picomolar range.

**
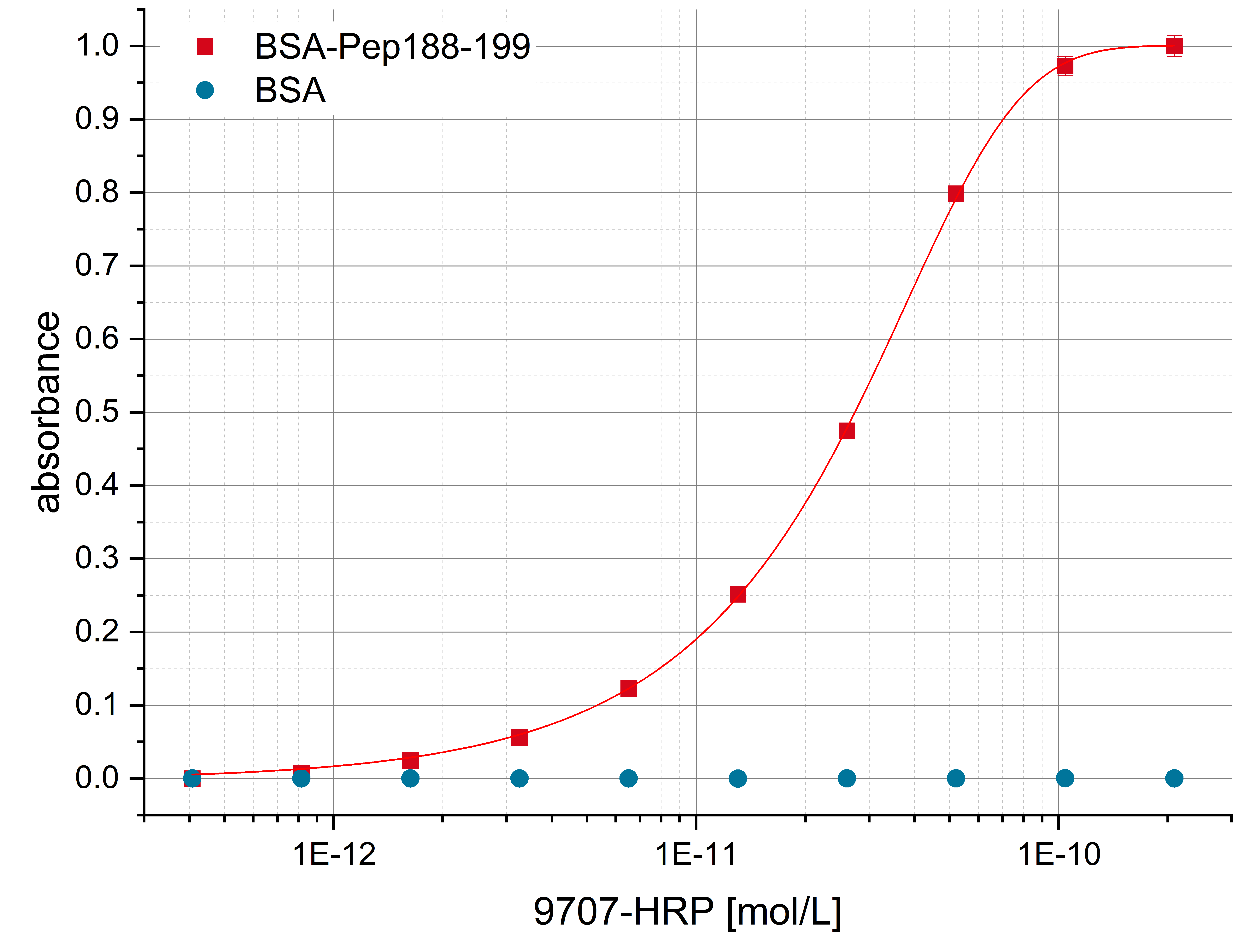
A**

**
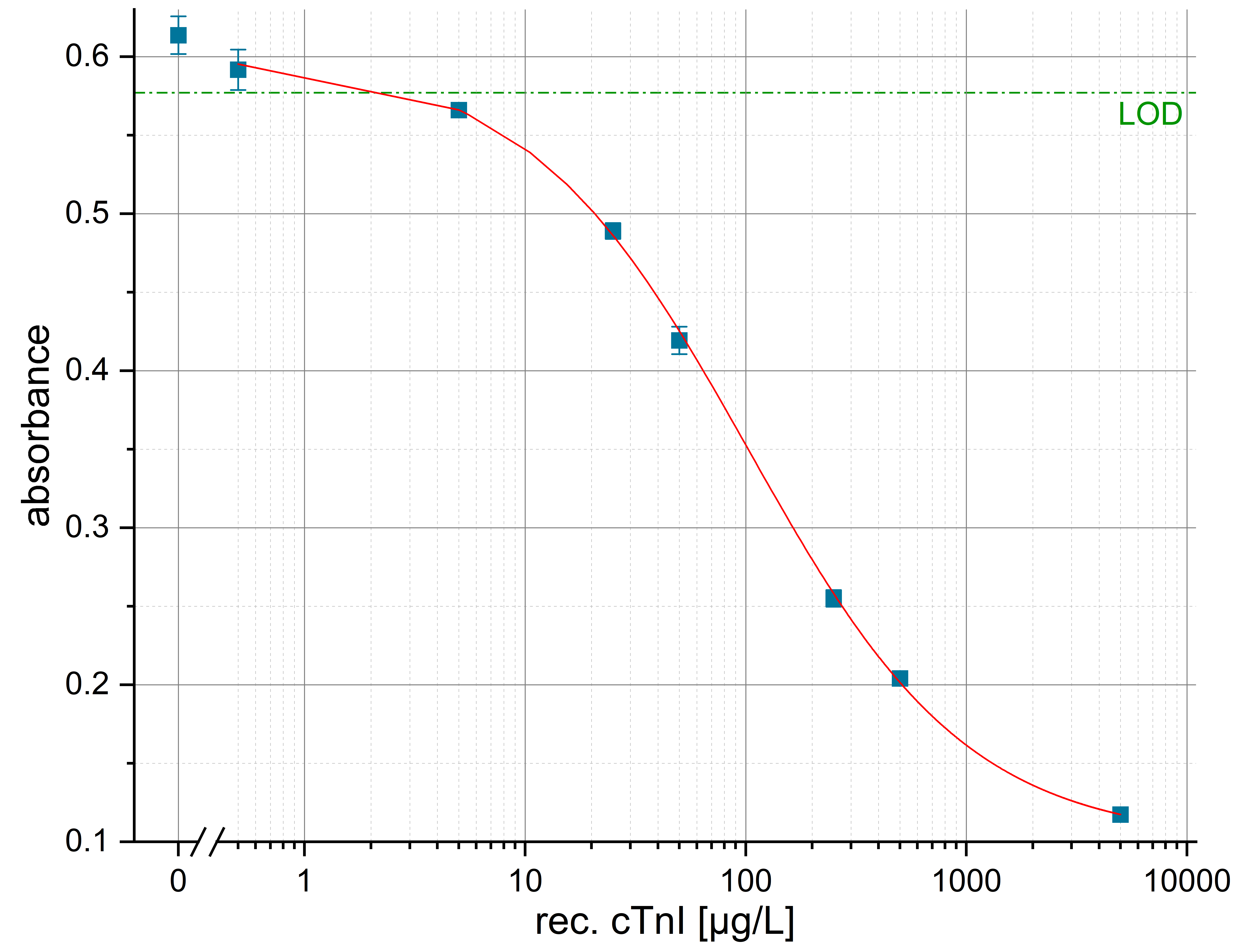
B**

**Figure S5.** 9707-HRP antibody binding against Pep188-199-SBAP-BSA conjugate (A) and 9707 antibody competition between the Pep188-199-SBAP-BSA conjugate and the expressed rec. cTnI (B).

The second approach was to test the antibody inhibition with the expressed recombinant cTnI (see figure S5, B). The peptide-BSA conjugate was immobilized in a microtiter plate and incubated with a constant concentration of 9707-HRP and an increasing concentration of recombinant cTnI as the analyte. As a result, an IC50 of 100 µg/L and a limit of detection of 2 µg/L could be achieved.

Biosensor and fluidic setup optimization

Test of CMOS sensor and temperature


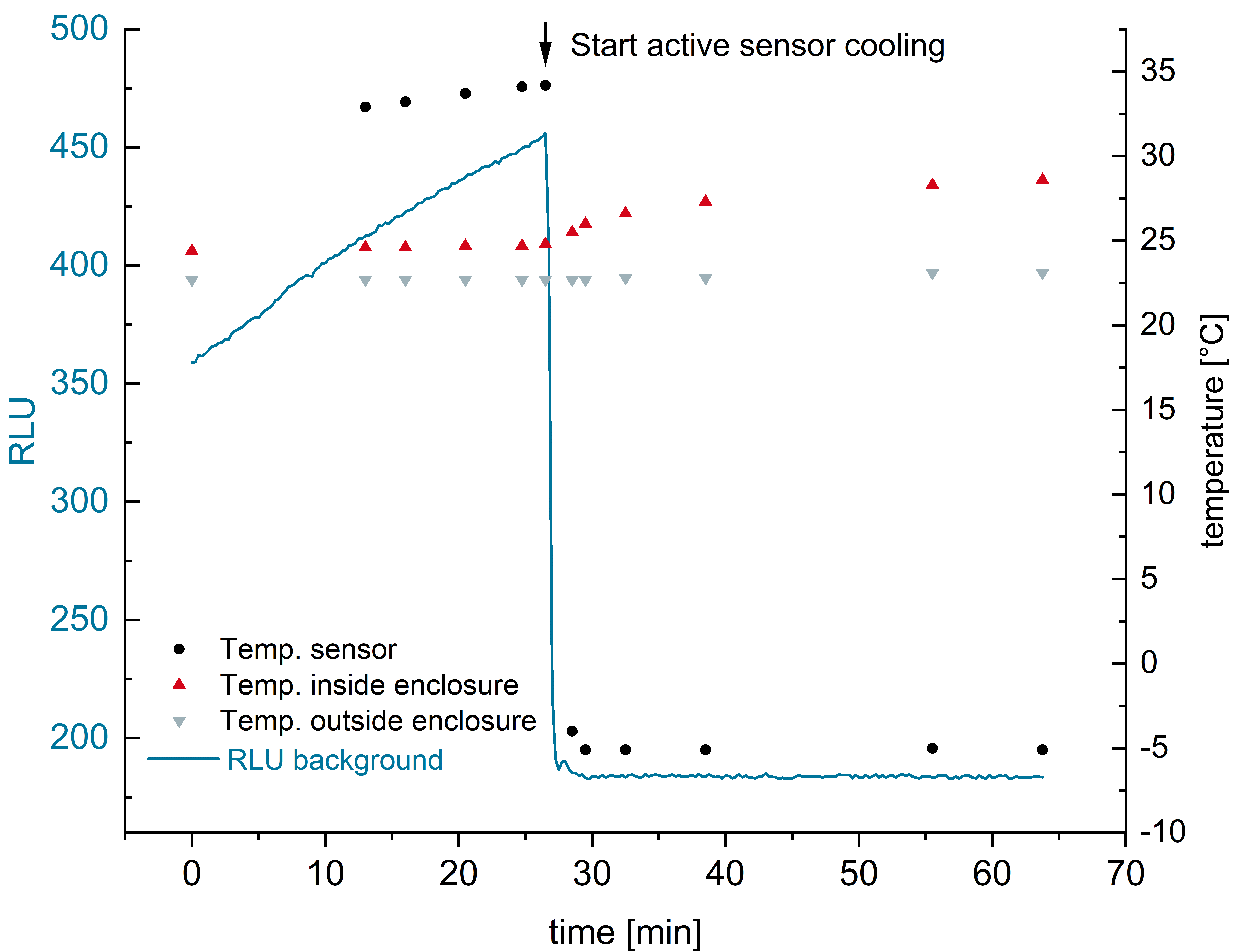
Enzymatically catalyzed chemiluminescence is a temperature-dependent reaction. Hence, the relative chemiluminescence (RLU) was measured depending on the sensor and environmental temperature (inside and outside the biosensor enclosure), as shown in figure S6.

**Figure S6.** Investigation of the noise behavior of the CMOS under various conditions, such as heat generation during measurement in and outside the enclosure after starting active cooling of the CMOS sensor to -5 °C. RLU was measured with an exposure of 15 sec.

Due to the operation of the CMOS sensor, the RLU increases within the first 25 minutes, as well as the sensor temperature. In order to achieve a low and constant chemiluminescent background signal, the sensor surface was actively cooled down to -5 °C. The temperature increase in the enclosure box was recorded as well. Usually, a CMOS cooling temperature of -5 °C and an environmental temperature equilibration of around 30 minutes was chosen before starting measurements.

Incubation time of chemiluminescence substrate


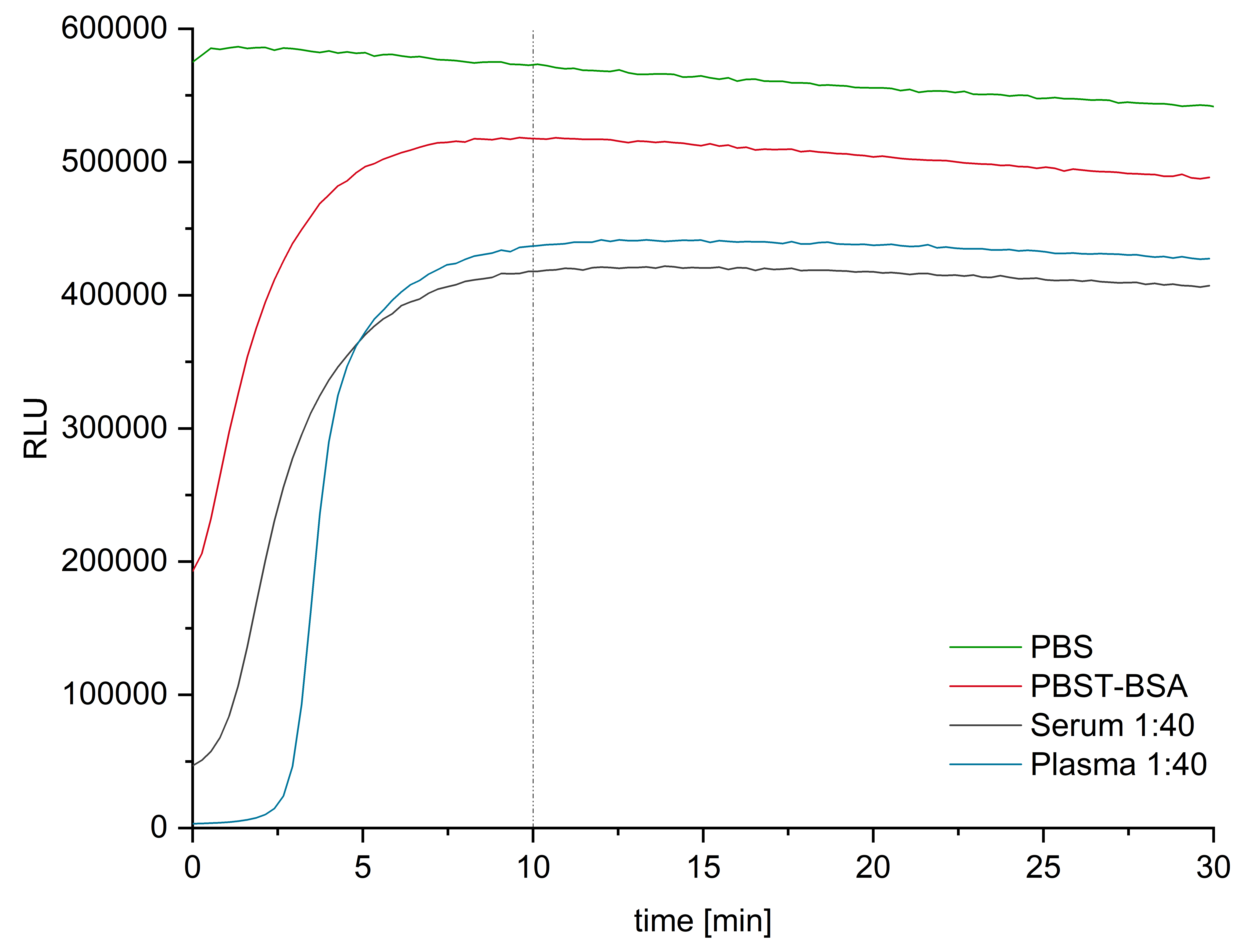
For the chemiluminescence substrate based on a mixture of luminol and H_2_O_2,_ the kinetics of signal development and its stability was investigated, as shown in Figure S7. The antibody-HRP conjugate was diluted to 1:60,000 in total, around 0.02 ng/L.

**Figure S7.** Relative luminescence unit (RLU) measurement of three different sample matrices (PBST-BSA, human serum 1:40, and human plasma 1:40) mixed with chemiluminescence substrate in a 1:1 ratio, simulating the biosensor measurement.

PBS shows an immediate signal rise, in contrast to the mixtures simulating a more complex sample matrix. The signal development of diluted serum and plasma reaches its maximum after around ten minutes. Therefore, an additional incubation loop was implemented.

Details of the biosensor setup


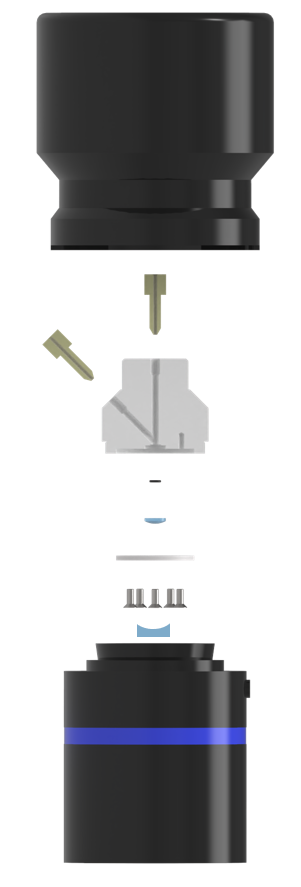

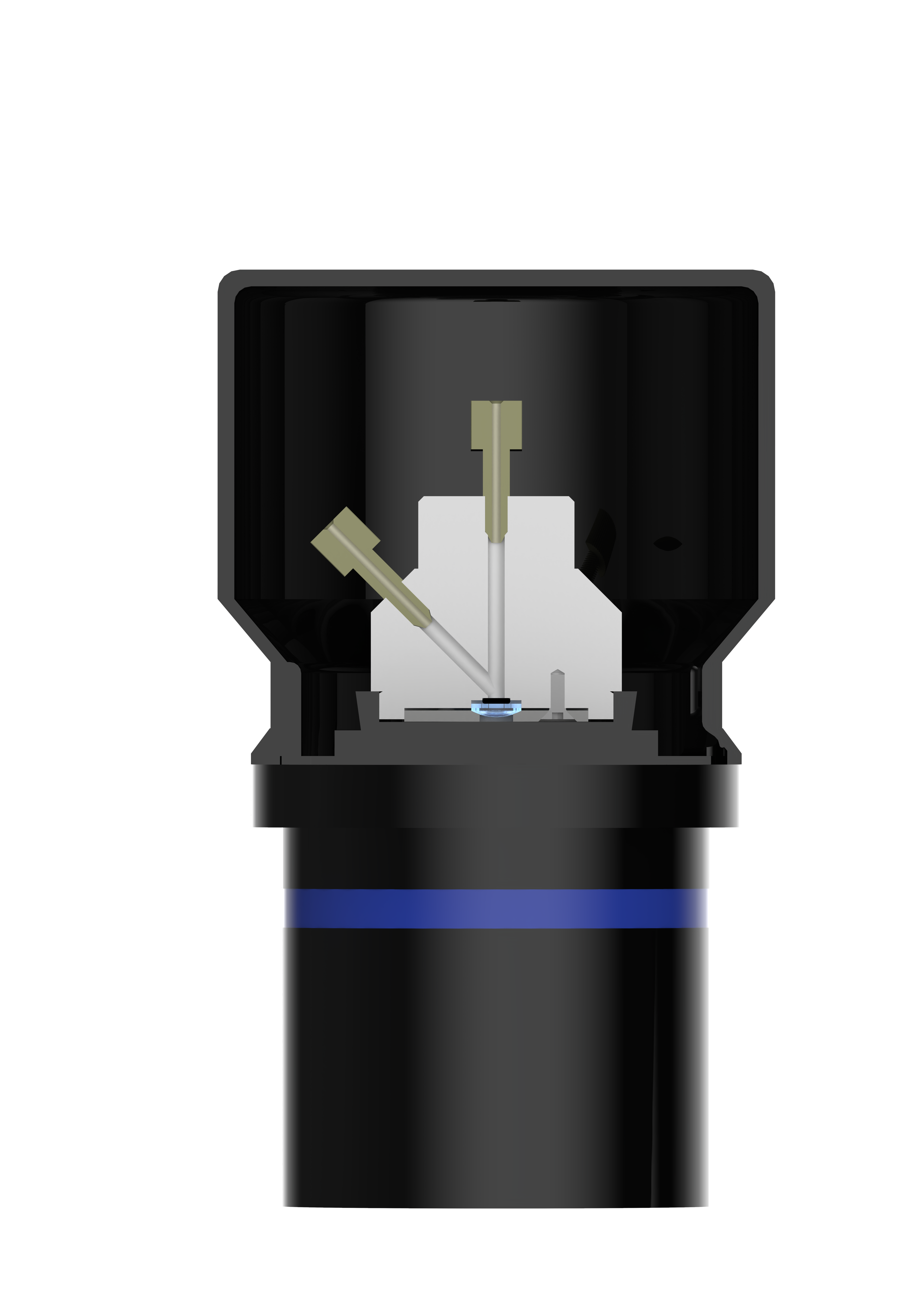

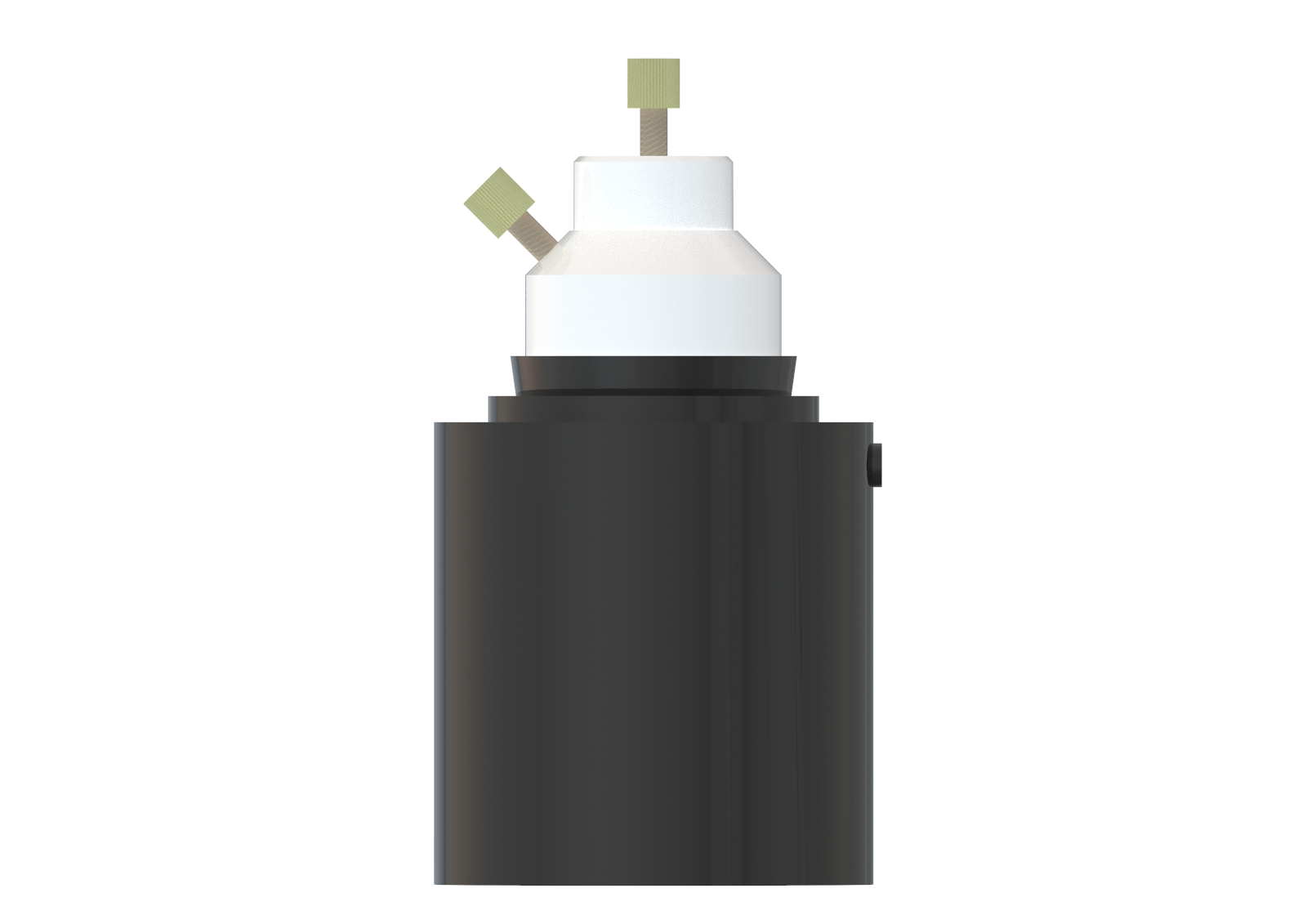


**
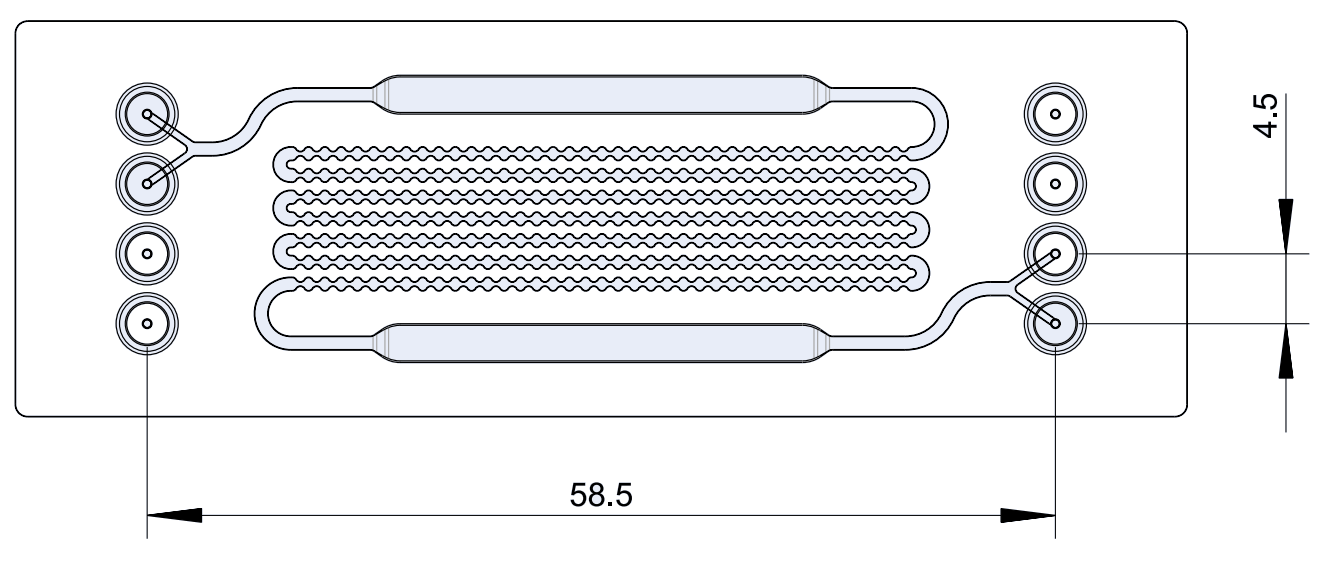
Figure S8.** Drawings of the flow cell made by subtractive manufacturing and the enclosure (1) made by additive manufacturing, including the CMOS camera setup (Solid Edge).

**Figure S9.** Drawing of the pearl-chain mixer chip (658) ordered from microfluidic ChipShop (Reproduced with courtesy of Microfluidic ChipShop GmbH).


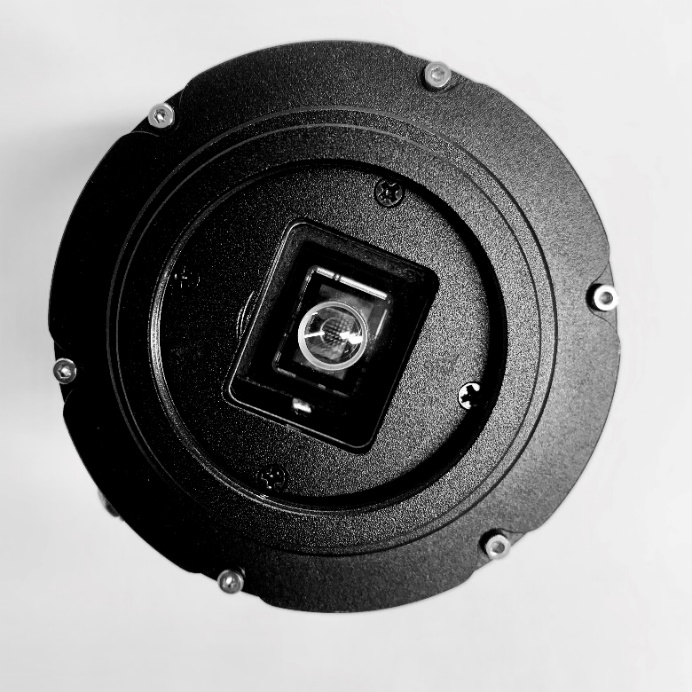
Optical readout of the biosensor

**
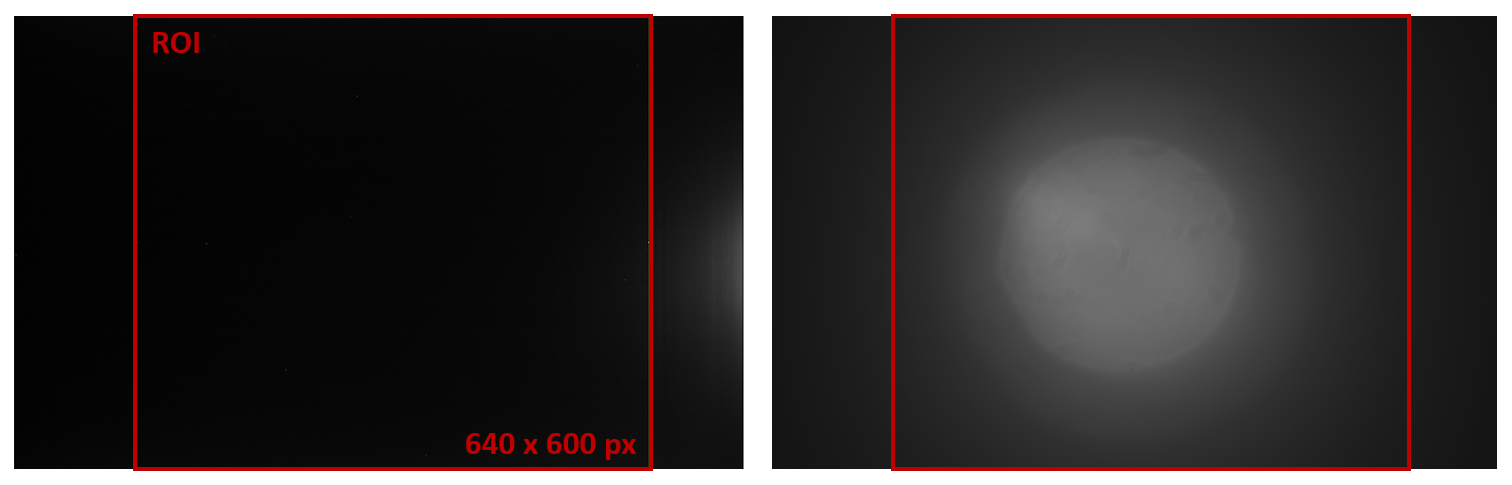
Figure S10.** Top view QHY 174 mono CMOS with a planoconcave lens (12 mm focal length, ∅ 9 mm) positioned on top of the protective glass of the separate CMOS chamber.

**Figure S11.** Images of the CMOS with 2x2 pixel binning resulting in an image size of 900x600 px. Highlighted region of interest (ROI, 640x600 px; ROI file for script.py can be found in the supplementary materials named: roi_region_x_y.txt, as well as the hot pixels file: hotpixels_960_600.txt for the biosensor measurements with an exposure time of 15 sec.

Measurement of recombinant cTnI


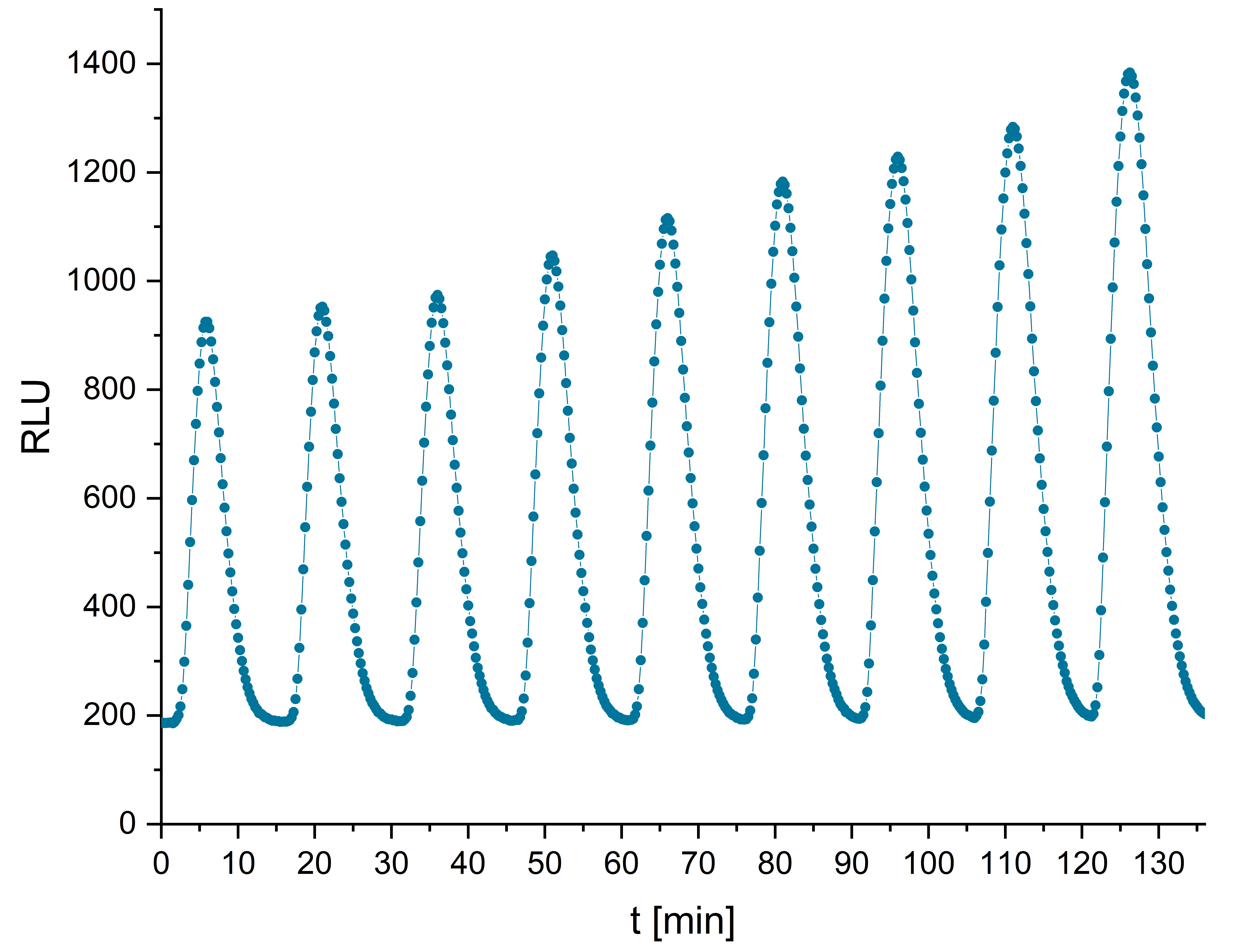


**Figure S12**. Calibration of recombinant troponin I in PBST-BSA in the lower concentration range. Injection of rec. cTnI in different concentrations: 0, 2, 4, 6, 8, 10, 12, 14, 16 µg/L.


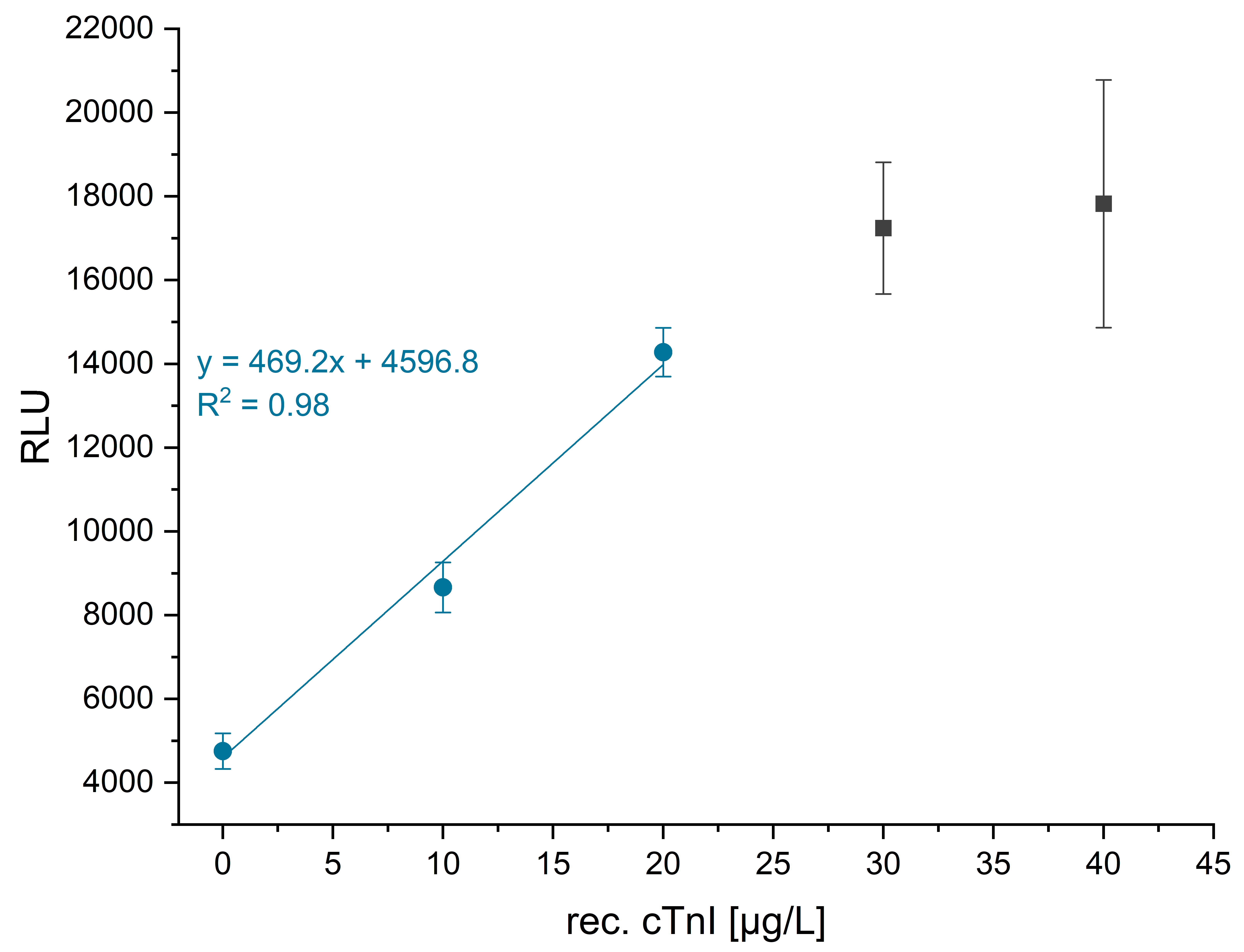


**Figure S13.** Exploration of the measurement range of recombinant cTnI between 0 and 40 µg/L.


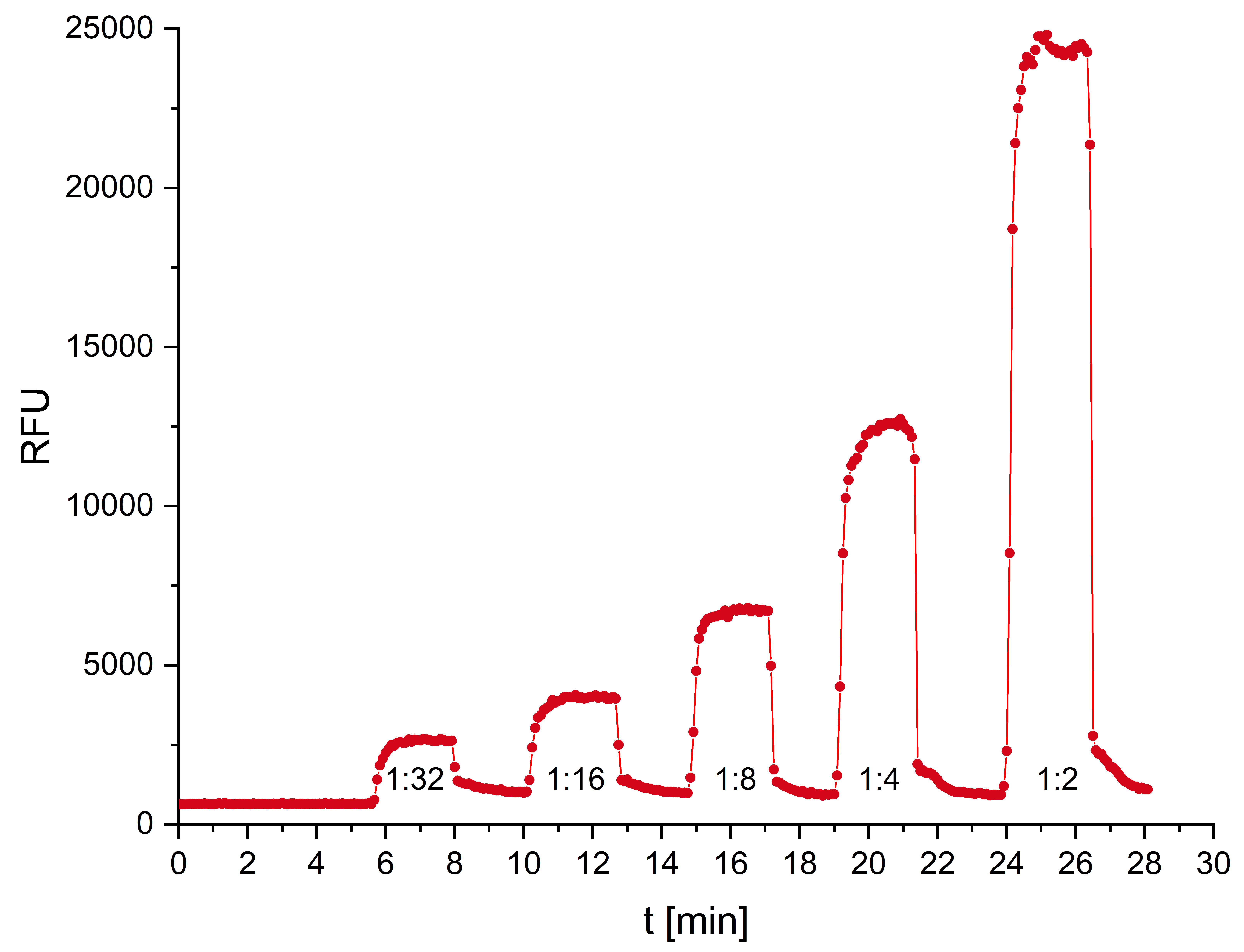
Matrix interference of blood plasma in fluorescence measurements (Excitation at 638 nm)

**Figure S13.** Background fluorescence measurement of blood plasma with the previously described biosensor setup [22]. These signals might be caused, e.g., by stray light or autofluorescence of the matrix. Lyophilized blood plasma was reconstituted, filtered, and diluted in ultra-pure water. 500 µL of different plasma dilutions were injected into the system via a six-way valve (without affinity column), and the signal was detected continuously (5 sec per frame). A semiconductor laser with a wavelength of approximately 638 nm was used. Two stacked long-pass filters and a dichroitic mirror were used to suppress light with a wavelength under 650 nm. The higher the concentration of blood plasma, the higher the values. This indicates a strong background signal.
